# Transcriptome-guided target identification of the TetR-like regulator SACE_5754 and engineered overproduction of erythromycin in *Saccharopolyspora erythraea*

**DOI:** 10.1186/s13036-018-0135-2

**Published:** 2019-01-24

**Authors:** Hang Wu, Zuling Chu, Wanxiang Zhang, Chi Zhang, Jingshu Ni, Heshi Fang, Yuhong Chen, Yansheng Wang, Lixin Zhang, Buchang Zhang

**Affiliations:** 1 School of Life Sciences, Institute of Physical Science and Information Technology, Anhui University, Hefei, 230601 China; 2 State Key Laboratory of Bioreactor Engineering, East China University of Science and Technology, Shanghai, 200237 China

**Keywords:** *Saccharopolyspora erythraea*, Erythromycin, TetR family transcriptional regulator, RNA-seq, SACE_5754, Overproduction

## Abstract

**Background:**

Erythromycin A (Er-A) produced by the actinomycete *Saccharopolyspora erythraea* is an important antibiotic extensively used in human medicine. Dissecting of transcriptional regulators and their target genes associated with erythromycin biosynthesis is crucial to obtain erythromycin overproducer strains through engineering of relevant regulatory elements in *S. erythraea*.

**Results:**

Here, we identified a TetR family transcriptional regulator (TFR), SACE_5754, negatively controlling erythromycin production. SACE_5754 indirectly repressed the transcription of *ery* cluster and cannot regulate itself and its adjacent gene *SACE_5753*. RNA-seq coupled with EMSAs and qRT-PCR was performed to identify the targets of SACE_5754, and confirmed that transcription of *SACE_0388* (encoding a pyruvate, water diknase), *SACE_3599* (encoding an antibiotic resistance macrolide glycosyltransferase) and *SACE_6149* (encoding a FAD-binding monooxygenase) were directly repressed by SACE_5754. A consensus palindromic sequence TYMAGG-n2/n4/n11-KKTKRA (Y: C/T, M: A/C, K: T/G, R: A/G) was proved to be essential for SACE_5754 binding using DNase I footprinting and EMSAs. During the three target genes of SACE_5754, *SACE_0388* and *SACE_6149* exhibited the positive effect on erythromycin production. Overexpression of either *SACE_0388* or *SACE_6149* in ∆*SACE_5754* further increased the Er-A production. By engineering the industrial strain *S. erythraea* WB with deletion of *SACE_5754* combined with overexpression of either *SACE_0388* or *SACE_6149*, Er-A production in WB*∆SACE_5754/*pIB139–0388 and WB*∆SACE_5754/*pIB139–6149 was successively increased by 42 and 30% compared to WB. Co-overexpression of *SACE_0388* and *SACE_6149* in WB*∆SACE_5754* resulted in enhanced Er-A production by 64% relative to WB. In a 5-L fermenter, WB*∆SACE_5754/*pIB139–0388-6149 produced 4998 mg/L Er-A, a 48% increase over WB.

**Conclusion:**

We have identified a TFR, SACE_5754, as a negative regulator of erythromycin biosynthesis, and engineering of *SACE_5754* and its target genes, *SACE_0388* and *SACE_6149*, resulted in enhanced erythromycin production in both wild-type and industrial *S. erythraea* strains. The strategy demonstrated here may be valuable to facilitate the manipulation of transcriptional regulators and their targets for production improvement of antibiotics in industrial actinomycetes.

**Electronic supplementary material:**

The online version of this article (10.1186/s13036-018-0135-2) contains supplementary material, which is available to authorized users.

## Background

Erythromycin A (Er-A) is widely used in clinic against pathogenic Gram-positive bacteria, industrially produced by the actinomycete *Saccharopolyspora erythraea* [1]. As a model of polyketide biosynthesis, erythromycin biosynthesis has been investigated by genetic and biochemical assays for production improvement and structure diversification [2]. At first, two primary metabolic precursors, propionyl-CoA and (2S)-methylmalonyl-CoA units, are enzymatically catalyzed by multifunctional modular PKS to form 6-deoxyerythronolide B (6-dEB) [3]. Next, a series of tailoring reactions including hydroxylation, glycosylation, and methylation take place to produce the final product Er-A. Erythromycin biosynthetic gene cluster (*ery* cluster) in *S. erythraea* contains 20 genes arranged in four major polycistronic units [4], but lacks regulatory genes, hampering efforts to improve erythromycin production by engineering relevant regulatory elements.

Traditionally, optimization of medium composition, random mutagenesis and selection have been performed to enhance erythromycin production [5]. Nowadays, rational engineering of the metabolic and regulatory pathways involved in biosynthesis of erythromycin was also an effective way to increase erythromycin production [6, 7]. Since the advent of “Omics” era, the genome-based functional investigations of antibiotic producers shed new lights on systematical understanding of molecular regulation of antibiotic biosynthesis [8]. Especially, transcriptome-driven reverse engineering strategy have been utilized for identifying and manipulating regulatory genes for production improvement of polyketide antibiotics [9]. However, few studies were reported on transcriptome-based target identification of transcriptional regulator associated with antibiotic biosynthesis in actinomycete.

The TetR family transcriptional regulators (TFRs), a class of regulators commonly found in bacteria, participated in diverse cellular processes [10]. In recent years, a variety of TFRs, inside or outside the gene cluster of antibiotic biosynthesis in actinomycete, were identified to regulate the biosynthesis of antibiotics by binding to the promoter region of their target genes [11–14]. Based on our previous prediction [12], 97 putative TFRs were encoded in the genome of *S. erythraea*, in which SACE_3986, SACE_7301, SACE_3446 and PccD (SACE_3396) were successively proved to regulate erythromycin biosynthesis [12, 15–17]. Nevertheless, additional TFRs associated with erythromycin production in *S. erythraea* need to be further identified to improve the understanding of regulatory mechanism underlying erythromycin biosynthesis.

Here, we identified a novel TFR, SACE_5754, indirectly repressing the erythromycin biosynthesis. SACE_5754 cannot regulate itself and its adjacent gene, increasing the difficulty to study its targets to understand the molecular mechanism of SACE_5754 for regulating erythromycin biosynthesis. Thereby, this study utilized RNA-seq based transcriptome analysis coupled with qRT-PCR, EMSAs and genetic experiments to identify and characterize SACE_5754’s targets related to erythromycin production. Further engineering *SACE_5754* and its target genes significantly increased erythromycin production in both wild-type and industrial *S. erythraea* strains.

## Results

### SACE_5754 plays a negative regulatory role in the biosynthesis of erythromycin

**Fig. 1:**
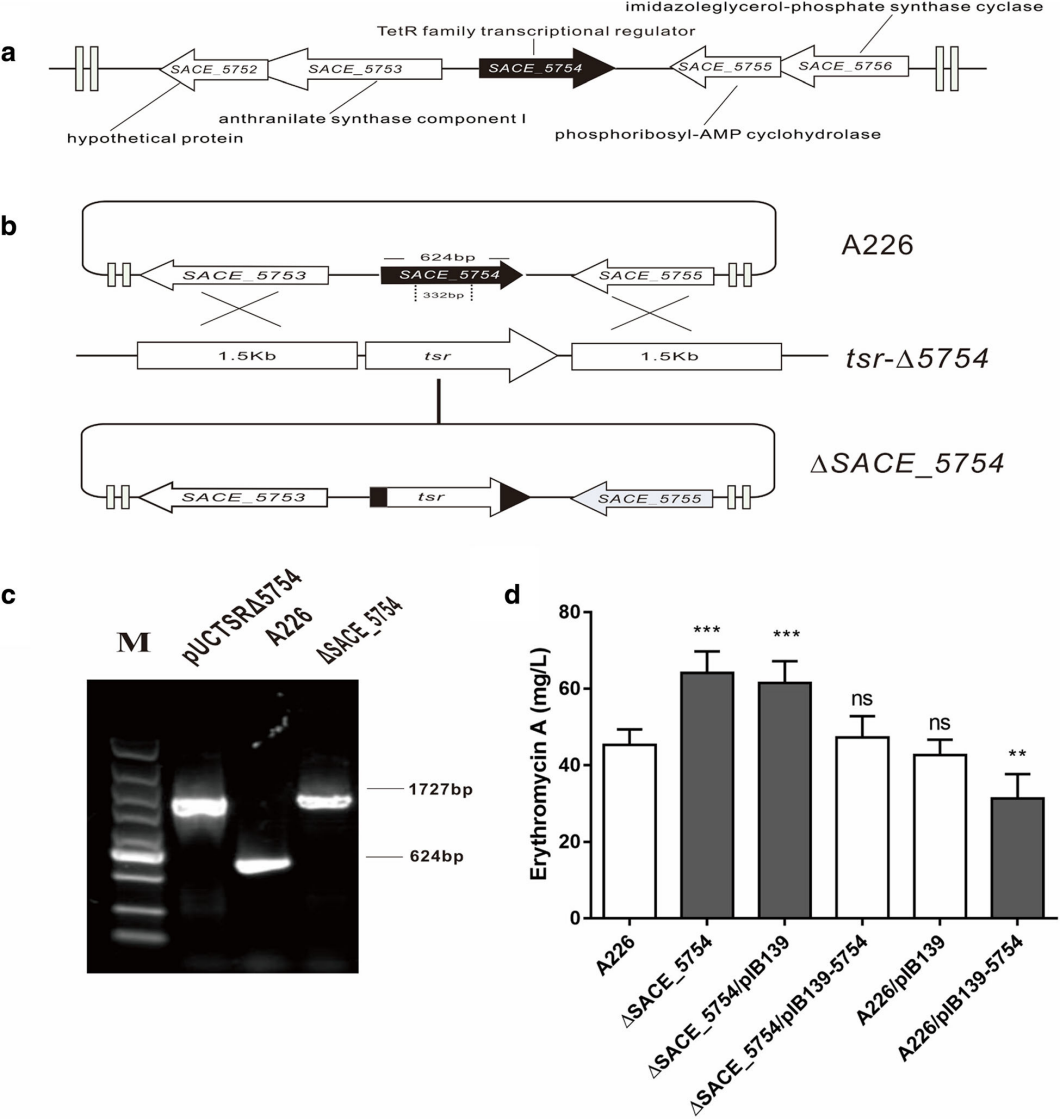
Inactivation of *SACE_5754* in *S. erythraea* A226. **a** Genetic organization of *SACE_5754* and its adjacent genes in *S. erythraea* A226. **b** Schematic deletion of *SACE_5754* by linearized fragment homologous recombination in *S. erythraea* A226. **c** PCR confirmation of the *SACE_5754* deletion mutant. Lanes: M, 5000-bp DNA ladder; pUCTSR∆*5754*, the positive control, 1727 bp amplified from pUCTSR∆*5754*; A226, the negative control, 624 bp amplified from A226; ∆*SACE_5754*, 1727 bp amplified from ∆*SACE_5754*. **d**. Er-A production in *S. erythraea* A226 and its derivatives. Mean values of three replicate are shown, with the standard deviation indicated by error bars. *p < 0.05, **p < 0.01, ***p < 0.001, ns, no significant (relative to A226)

Considering the key role of TFRs in antibiotic biosynthesis in actinomycetes, we performed gene inactivation and identified several TFRs involved in erythromycin production in *S. erythraea*. These contained three TFRs (SACE_3986, SACE_7301 and SACE_3446) previously published and SACE_5754 currently investigated. *SACE_5754* contains 624 nucleotides with approximately 22 kDa molecular mass. The location of *SACE_5754* and its adjacent genes on the chromosome were shown in Fig. 1a. Blast analysis revealed that SACE_5754 homologs are widely distributed among actinomycetes, suggesting that this TFR may have important biological functions in actinomycetes (Additional file 1: Figure S1a).

*SACE_5754* was disrupted with a *tsr* replacement in *S. erythraea* A226, generating the ∆*SACE_5754* mutant (Fig. 1b and c). Er-A production in ∆*SACE_5754* (64 mg/L) was 41% higher than that in strain A226 (45 mg/L). The complemented strain ∆*SACE_5754*/pIB139–5754 restored the original erythromycin level, suggesting that production enhancement of erythromycin in ∆*SACE_5754* was solely due to the *SACE_5754* disruption (Fig. 1d). Furthermore, pIB139–5754 and pIB139 was individually introduced into strain A226. The *SACE_5754*-overexpressed strain A226/pIB139–5754 (31 mg/L) successively exhibited 31 and 28% reduction in Er-A production relative to A226 and A226/ pIB139 (43 mg/L) (Fig. 1d). Deletion and overexpression of *SACE_5754* in strain A226 had no effect on cell growth and sporulation, inferring that SACE_5754 was not involved in cell growth or morphological differentiation of *S. erythraea* (Additional file 1: Figure S1b and 1c).

### SACE_5754 indirectly regulates erythromycin biosynthesis

**Fig. 2:**
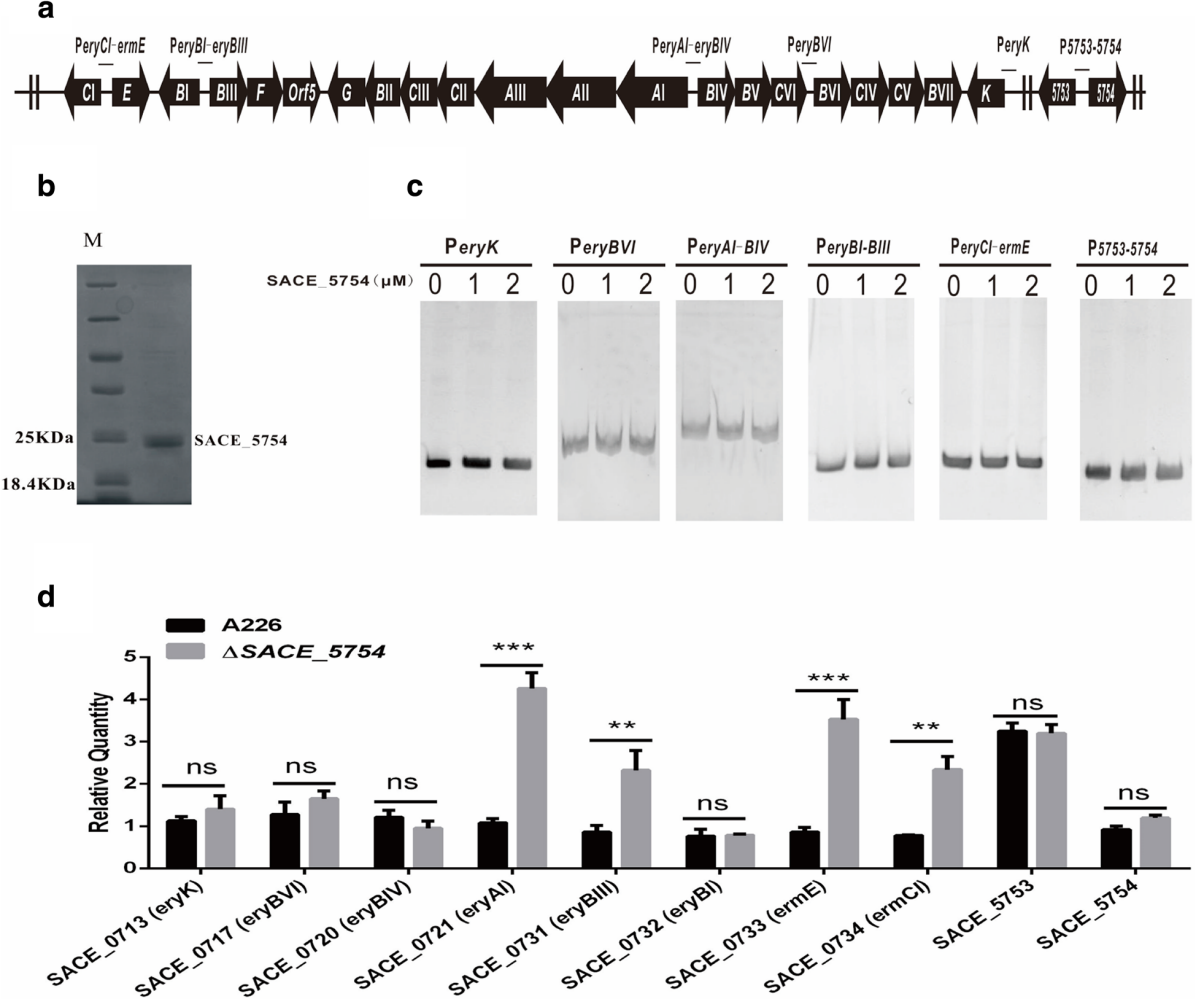
SACE_5754 negatively regulates erythromycin production in *S. erythraea* A226. **a** Genetic organization of *ery* cluster, *SACE_5754* and its adjacent gene *SACE_5753* in *S. erythraea* A226. **b** Purification of His_6_-tagged SACE_5754. **c** EMSAs of the interaction of probes P_*eryBVI*_, P_*eryAI-BIV*_, P_*eryBI-BIII*_, P_*eryCI-ermE*_, P_*eryK*_ and P_*5753–5754*_ with purified His_6_-SACE_5754 protein. **d** Effect of *SACE_5754* deletion on transcription levels of *ery* cluster, *SACE_5753* and *SACE_5754.* qRT-PCR was used to quantify the amounts of transcripts in A226 and Δ*SACE_5754* cultured for 48 h in liquid R5 medium. Mean values of three replicates are shown, with the standard deviation indicated by error bars.*p < 0.05, **p < 0.01, ***p < 0.001, ns, no significant

To prove the possibility that SACE_5754 regulates erythromycin production through *ery* cluster (Fig. 2a), we measured transcripts of *ery* cluster by qRT-PCR. Results showed that the transcriptional levels of *eryAI* (SACE_0721, encoding polyketide synthase I), *eryBIII* (SACE_0731, encoding NDP-4-keto-2, 6-dideoxyhexose 3-C-methyltransferase), *ermE* (SACE_0733, encoding rRNA methyltransferase) and *eryCI* (SACE_0734, encoding transaminase) were respectively increased by 4-, 2.7-, 4- and 3- folds compared with A226 (Fig. 2d), indicating SACE_5754 might negatively regulate erythromycin biosynthesis.

To examine whether SACE_5754 might directly regulate transcription of *ery* cluster, it was expressed in *E. coil* BL21 (DE3), and was used to examine its affinity to the five regions containing *ery* promoters with EMSAs (Fig. 2b). Results showed that SACE_5754 did not bind to *ery* promoters (Fig. 2c), demonstrating that SACE_5754 may regulate erythromycin production by indirectly repressing the expression of *ery* cluster.

### SACE_5754 cannot transcriptionally regulate its own gene and its adjacent gene *SACE_5753*

Most TFRs are transcriptional regulators to regulate their adjacent genes and/or themselves [6]. To investigate whether SACE_5754 directly binds to its adjacent gene *SACE_5753* and to its own promoter regions, His_6_-SACE_5754 was used to perform EMSAs with DNA fragment P_*5753–5754*_, which covers the entire promoter regions of *SACE_5753* and *SACE_5754*, but no obvious gel shift band was detected. Furthermore, we measured transcripts of *SACE_5754* and *SACE_5753* by qRT-PCR, and found that the transcriptional levels of *SACE_5754* and *SACE_5753* had no change in A226 and ∆*SACE_5754*, suggesting that SACE_5754 cannot regulate its own gene and adjacent gene *SACE_5753* (Fig. 2d).

### Finding SACE_5754 target genes based on transcriptomic data

**Fig. 3:**
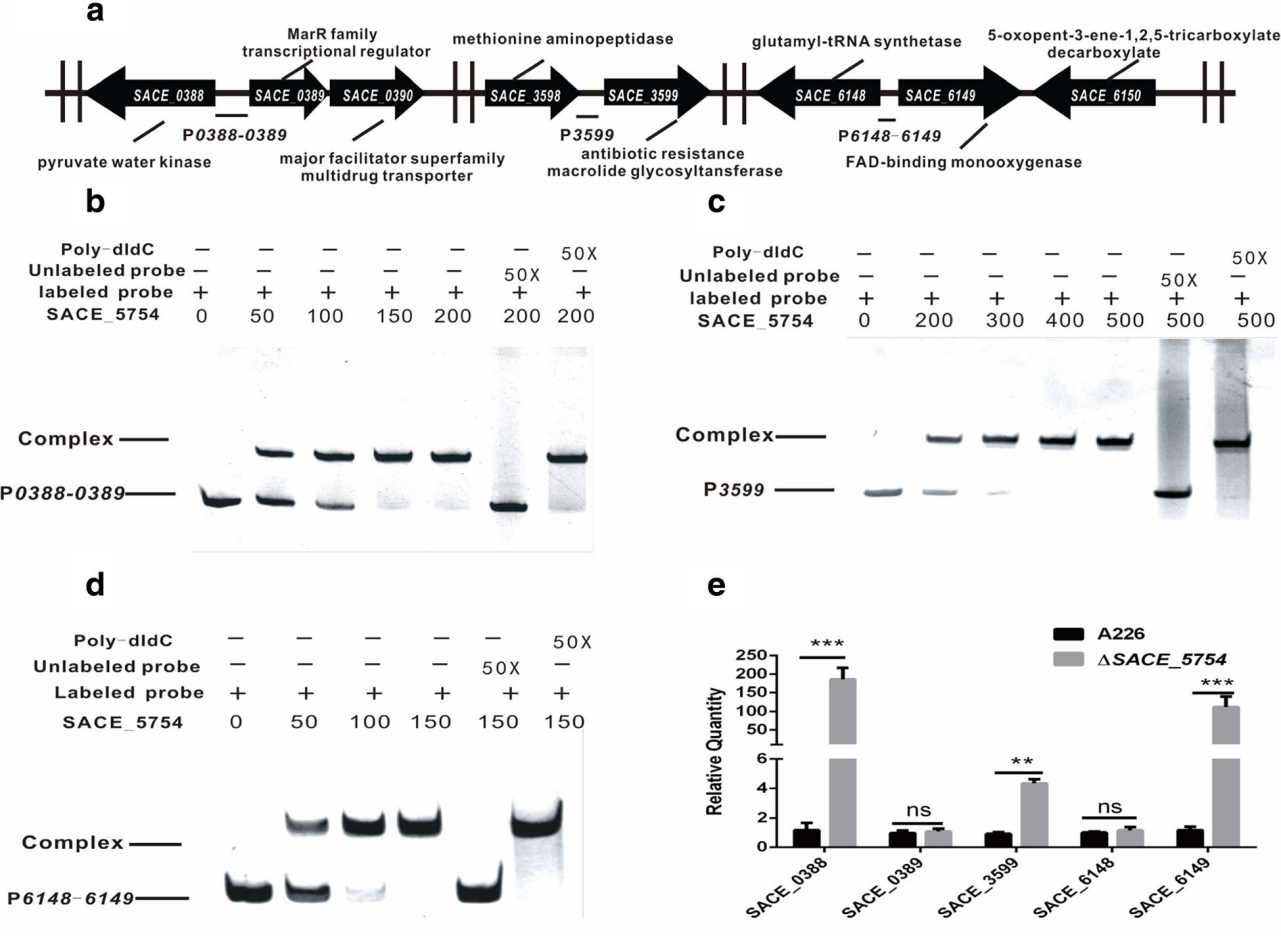
SACE_5754 directly represses the expression of *SACE_0388*, *SACE_3599* and *SACE_6149*. **a** Genetic organization of candidate target genes of SACE_5754. **b** EMSAs with SACE_5754 and P_*0388–0389*_. **c** EMSAs with SACE_5754 and P_*3599*_. **d** EMSAs with SACE_5754 and P_*6148–6149*_. **e** Effect of *SACE_5754* deletion on transcription levels of related genes. qRT-PCR was used to quantify the amounts of transcripts in A226 and Δ*SACE_5754* cultured for 48 h in liquid R5 medium**.** Mean values of three replicates are shown, with the standard deviation indicated by error bars. *p < 0.05, **p < 0.01, ***p < 0.001, ns, no significant

In order to search for SACE_5754’s targets, transcriptomic comparison between A226 and ∆*SACE_5754* was performed by RNA-seq. With the statistic criteria of Foldchange ≥2 and probability ≥0.8, a total of 623 differential expressed genes were identified (440 up-regulated genes and 183 down-regulated genes) in ∆*SACE_5754*. Herein, promoter regions of 80 genes with a greater than 0.9 probability in the transcriptomic data were tested by EMSAs (Additional file 1: Table S3), and results showed that SACE_5754 could specifically bind to P_*0388–0389*_、P_*3599*_ and P_*6148–6149*_ (Fig. 3a, b and c). Further qRT-PCR analysis found that *SACE_0389* and *SACE_6148* were not affected by *SACE_5754* deletion, but the transcriptional levels of *SACE_0388*, *SACE_3599* and *SACE_6149* were increased by 158-, 4.7- and 95- folds, respectively (Fig. 3d). It seemed that SACE_5754 directly represses the transcription of *SACE_0388*, *SACE_3599* and *SACE_6149*.

### Identification of the SACE_5754-binding site

**Fig. 4:**
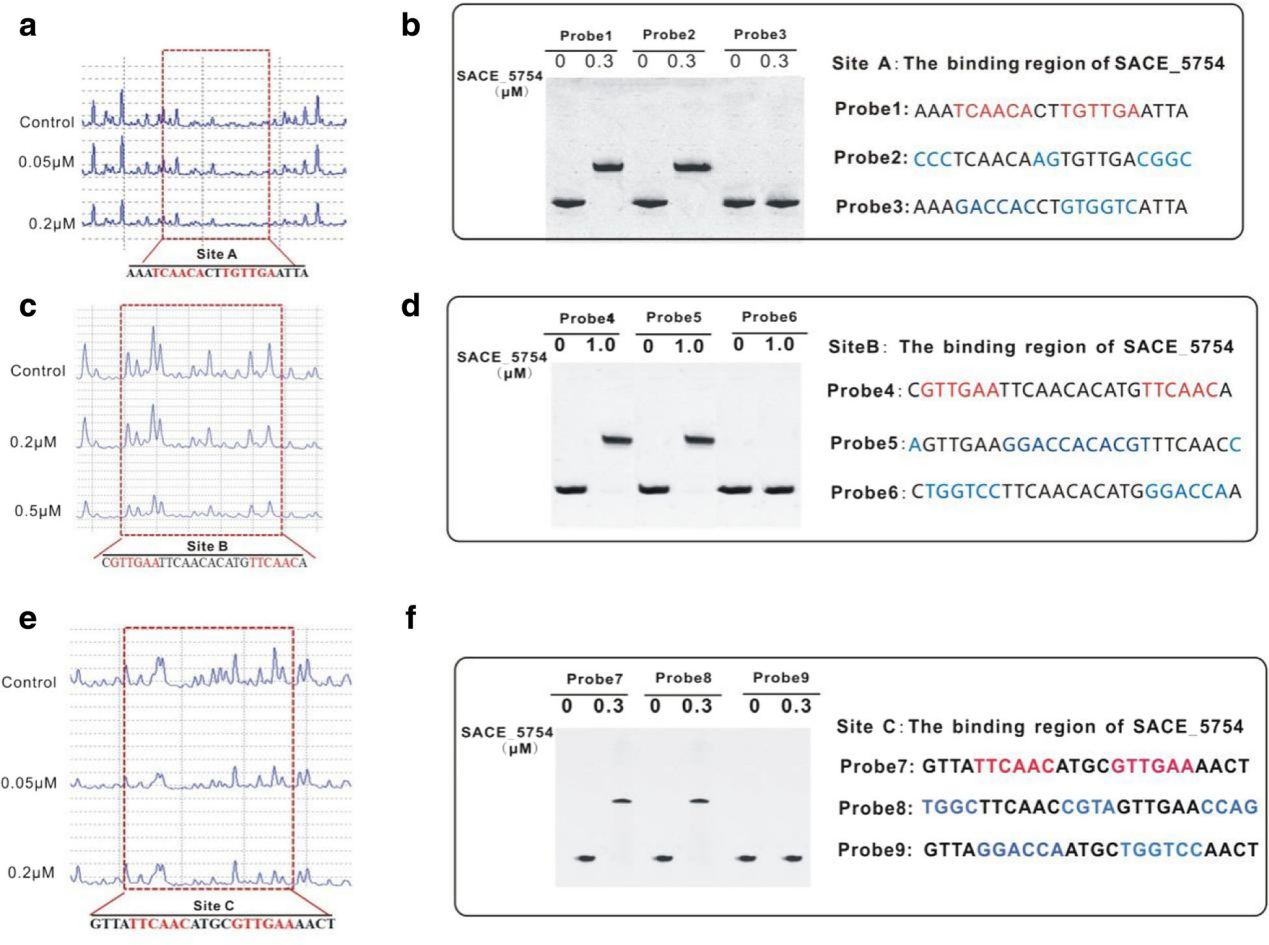
Analysis of the SACE_5754-binding sites. **a** DNase I footprinting assay of SACE_5754-binding site in the P_*0388–0389*_. Upper fluorogram: control reaction without protein. Protection regions were acquired with increasing concentrations (0.5 μM and 0.2 μM) of His_6_-SACE_5754 protein. **b** SACE_5754 binds to the probe P1and mutated probe P2 and P3 by EMSAs. **c** DNase I footprinting assay of SACE_5754-binding site in the P_*3599*_. Upper fluorogram: control reaction without protein. Protection regions were acquired with increasing concentrations (0.2 μM and 0.5 μM) of His_6_-SACE_5754 protein. **d** SACE_5754 binds to the probe P4 and mutated probe P5 and P6 by EMSAs. **e** DNase I footprinting assay of SACE_5754-binding site in the P_*6148–6149*_. Upper fluorogram: control reaction without protein. Protection regions were acquired with increasing concentrations (0.05 μM and 0.2 μM) of His_6_-SACE_5754 protein. **f** SACE_5754 binds to the probe P5 and mutated probe P6 and P7 by EMSAs

In order to precisely determine SACE_5754’s binding site within the intergenic region of *SACE_0388* and *SACE_0389*, a DNase I footprinting experiment was performed, and a 12-bp palindromic sequence AAATCAACACTTGTTGAATTA (termed site A) in P_*0388–0389*_ was protected by SACE_5754 (Fig. 4a). To assess the importance of the palindromic sequence for SACE_5754 binding, EMSAs were performed with different probes, named probe 1 (no mutation of site A), probe 2 (with base substitution without the palindromic sequence of site A) and probe 3 (with base substitution within the palindromic sequence of site A) (Fig. 4b). As expected, probe 2 showed the same band shift as probe 1, while probe 3 showed no binding (Fig. 4b), demonstrating that TCAACA-n2-TGTTGA was indispensable for SACE_5754 binding.

DNase I footprinting experiments showed that P_*3599*_ and P_*6148–6149*_ also had 12-bp palindromic sequences protected by SACE_5754, CGTTGAATTCAACACATG TTCAACA (termed site B) and GTTATTCAACATGCGTTGAAAACT (termed site C), respectively (Fig. 4c and e). EMSAs with base substitution mutagenesis showed that the 12-bp palindromic sequences were indispensable for SACE_5754 binding (Fig. 4d and f). Those findings indicated that the palindromic sequence TYMAGG-n2/n4/n11-KKTKRA (Y: C/T, M: A/C, K: T/G, R: A/G) was essential for SACE_5754 binding.

### Overexpression of *SACE_0388* and *SACE_6149* in ∆*SACE_5754* improves erythromycin production

**Fig. 5:**
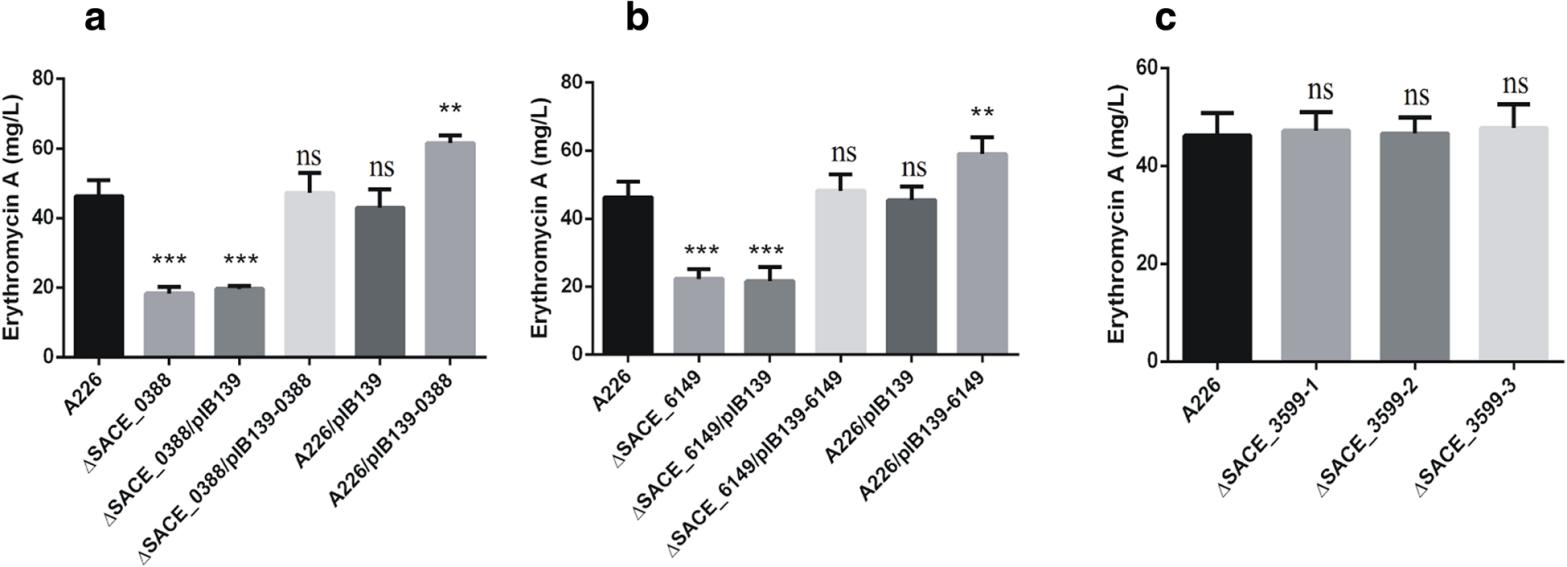
Relationship of *SACE_0388*, *SACE_3599* and *SACE_6149* with erythromycin production. **a** Er-A production in *S. erythraea* A226 and *SACE_0388* mutant strains. **b** Er-A production in *S. erythraea* A226 and *SACE_6149* mutant strains. **c** Er-A production in A226, *SACE_3599*-deleted strains ∆*SACE_3599*–1, ∆*SACE_3599*–2 and ∆*SACE_3599*–3*.* Mean values of three replicate are shown, with the standard deviation indicated by error bars. *p < 0.05, **p < 0.01, ***p < 0.001, ns, no significant. (relative to A226)

In order to explore the relationship of *SACE_0388*, *SACE_3599* and *SACE_6149*, SACE_5754’s targets, to erythromycin biosynthesis, the ∆*SACE_0388*, ∆*SACE_3599* and ∆*SACE_6149* mutants were individually constructed (Additional file 1: Figure S2-S4). Compared with A226, ∆*SACE_0388* and ∆*SACE_6149* respectively produced lower Er-A by 80 and 60% (Fig. 5a and b); however, ∆*SACE_3599* showed no yield change (Fig. 5c). Overexpression of *SACE_0388* and *SACE_6149* in A226 improved Er-A production by 32 and 28% (Fig. 5a and b). Those findings indicated that SACE_0388 and SACE_6149 had the positive effect on erythromycin production, while SACE_3599 did not affect erythromycin production.

**Fig. 6:**
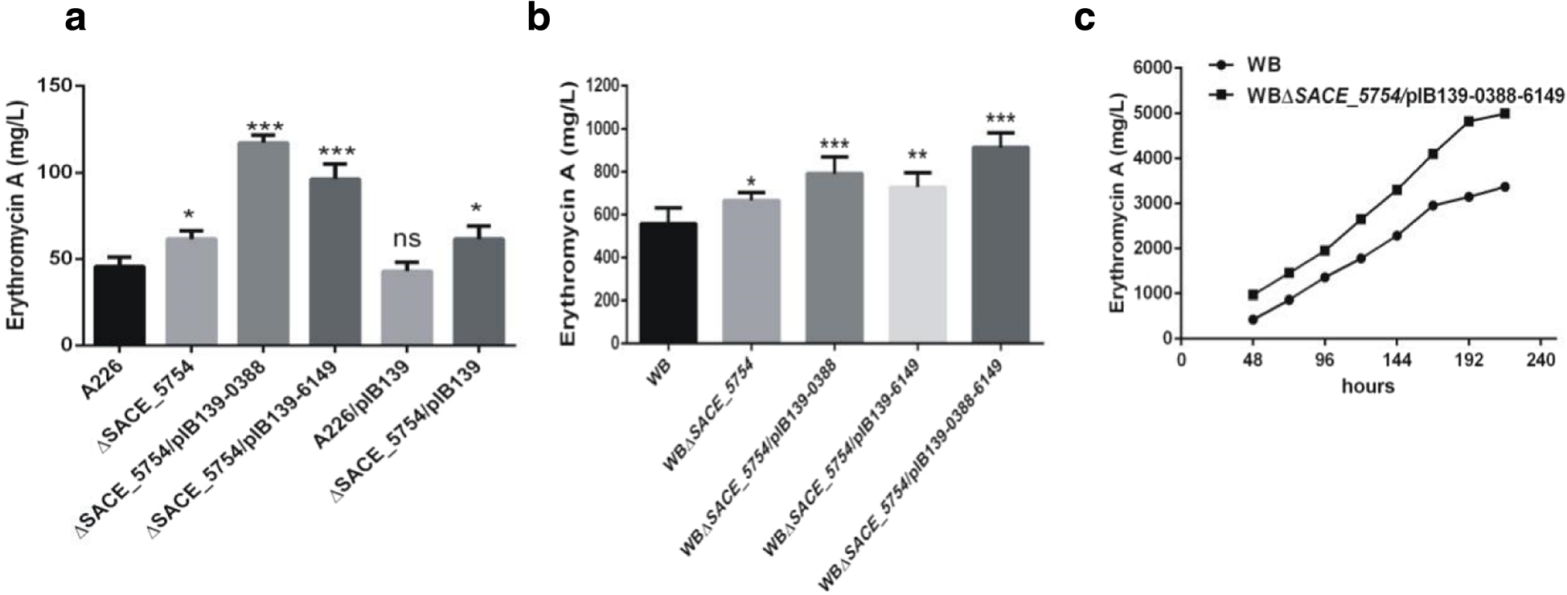
Using SACE_5754 pathway to improve erythromycin production. **a** Er-A production in A226 and its derivatives. **b** Er-A production in *S. erythraea* WB and its derivatives. Mean values of three replicate are shown, with the standard deviation indicated by error bars. *p < 0.05, **p < 0.01, ***p < 0.001, ns, no significant (relative to A226 or WB). **c** Time course of Er-A production of WB and WB*∆SACE_5754 /*pIB139–0388-6149 in a 5 L fermenter

Furthermore, to investigate whether overexpression of *SACE_0388* and *SACE_6149* in ∆*SACE_5754* would further affect Er-A production, pIB139–0388 and pIB139–6149 was introduced into ∆*SACE_5754* to obtain ∆*SACE_5754*/ pIB139–0388 and ∆*SACE_5754*/pIB139–6149, respectively. Compared with ∆*SACE_5754*, ∆*SACE_5754*/ pIB139–0388 and ∆*SACE_5754*/pIB139–6149 had 83 and 50% increase in the Er-A production, respectively (Fig. 6a).

### Engineering high-producing industrial *S. erythraea* WB enhances erythromycin production

To evaluate the possible improvement of erythromycin production in industrial settings, SACE_5754 and its target genes *SACE_0388* and *SACE_6149* were engineered in a high-yield industrial *S. erythraea* WB. Firstly, when *SACE_5754* was deleted in WB, the obtained WB*∆SACE_5754* exhibited production improvement of Er-A by 19% (Fig. 6b). Secondly, either pIB139–0388 or pIB139–6149 was introduced into WB*∆SACE_5754*. Compared with WB, the resulted WB*∆SACE_5754/*pIB139–0388 and WB*∆SACE_5754/*pIB139–6149 improved Er-A production by 42 and 30%, respectively (Fig. 6b). Lastly, co-expression of *SACE_0388* and *SACE_6149* in WB*∆SACE_5754* led to a 64% increase in Er-A production compared to WB (Fig. 6b). Considering further application of the engineered strain in industry, we cultured WB∆SACE_5754/pIB139–0388-6149 in a 5-L fermenter for time course analysis of erythromycin production. The production of Er-A was increased by 48% from 3327 mg/L in WB to 4998 mg/L in WB∆*SACE_5754*/pIB139–0388-6149 (Fig. 6c).

## Discussion

TFRs can be grouped into three types based on their orientation relative to neighboring genes in the genome, which can be used to predict the target genes of TFRs [10]. Type I TFR is divergently oriented to a neighboring transcription unit, as is the case for *tetR* and *tetA*, where the intergenic region between the two genes is less than 200 bp [18]. The majority of TFR genes are type I TFRs, which are generally auto-regulatory and also control the expression of adjacent genes [10]. The present work has characterized a novel TFR, SACE_5754, which negatively controls erythromycin biosynthesis in *S. erythraea*. However, SACE_5754, a type I TFR, cannot regulate itself and *SACE_5753*, and also didn’t bind to promoter regions of *ery* cluster, hampering the effort to identify its targets for mechanistic dissection of SACE_5754 regulating erythromycin biosynthesis. In this study, RNA-seq was first used to perform the transcriptomic comparison between the parental strain A226 and the ∆*SACE_5754* mutant. Overall, 440 genes were up-regulated while 183 genes were down-regulated in ∆*SACE_5754*, with most of the genes related to the cellular metabolism in *S. erythraea*. Further, promoter regions of 80 genes with a greater than 0.9 probability in the RNA-seq data were selected by EMSAs to search for SACE_5754’s targets. Our results proved that SACE_5754 directly repressed the expression of *SACE_0388*, *SACE_3599* and *SACE_6149* by binding to the palindromic sequence TYMAGG-n_2_/n_4_/n_11_-KKTKRA (Y: C/T, M: A/C, K: T/G, R: A/G).

*SACE_0388*, encoding a pyruvate, water diknase, catalyze pyruvate into phosphoenol-pyruvate, which may enter into TCA cycle. Methylmalonyl-CoA can either be filled or drained by methylmalonyl-CoA mutase-catalyzed reaction converting intermediate succinyl-CoA and methylmalonyl-CoA of TCA cycle in *S. erythraea* [19, 20]. Coupled with transcriptome data, the expression of several genes involved in TCA cycle (citrate synthase, *SACE_0069*; fumarate reductase iron-sulfur subunit, *SACE_1171*; malate dehydrogenase, *SACE_3674*; methylmalonyl-CoA mutase subunit beta, *SACE_5638*; methylmalonyl-CoA mutase, *SACE_5639*; succinyl-CoA synthetase subunit alpha, *SACE_6668*; succinyl-CoA synthetase subunit beta, *SACE_6669*; phosphoenolpyruvate carboxykinase, *SACE_7274*) were analyzed by qRT-PCR. Compared to A226, *SACE_1171* was transcriptionally down-regulated by 2.4- folds while the transcription of *SACE_7274* was up-regulated by 2.0- folds, and other genes was not transcriptionally regulated by *SACE_5754* (Additional file 1: Figure S5). These results suggested that *SACE_0388* and *SACE_ 7274* may carry more phosphoenol-pyruvate to TCA cycle while *SACE_1171* may reduce the flow of succinyl-CoA to succinyl but instead converts to methylmalony-CoA metabolite pool, suggesting a possible way of enhancing erythromycin yield. Considering that *SACE_0389*, encoding a MarR family transcriptional regulator, was not differentially transcribed upon *SACE_5754* inactivation, we did not perform gene inactivation of *SACE_0389*.

SACE_3599 encodes an antibiotic resistance macrolide glycosyltransferase. Glycosyltransferase is an important enzyme that catalyzes the attachment of sugar moieties to acceptor molecules [21]. Deletion of *SACE_3599* in A226 didn’t affect erythromycin production, and transcription of *SACE_3599* was increased by ~ 4-folds in ∆*SACE_5754* relative to that in A226. Recent investigation found that through the regulation of a protein-level modification with overexpression of phosphopantetheinyl transferase genes in 33 *Actinomycetes*, most of strains displayed novel metabolite profiles [22]. We speculated that that SACE_3599 might be responsible for the biosynthesis of other secondary metabolites, which is worthwhile to do follow-up exploration. SACE_6149, encoding a FAD-binding monooxygenase which has been shown to cover a wide range of different oxygenation reactions [23], may modify some intermediates in erythromycin synthesis. Detailed insights of *SACE_6149* into erythromycin production require further investigation. Based on current results, we propose a pathway of regulation of erythromycin production by SACE_5754 in *S. erythraea* (Additional file 1: Figure S6).

SACE_5754 stimulates erythromycin production by altering the transcription of *ery* cluster. However, this effect is indirect and SACE_5754’s downstream regulator interacting with promoter regions of *ery* cluster in *S. erythraea* remains unknown. Several transcriptional regulators were previously reported to involve in erythromycin biosynthesis in *S. erythraea*. For example: DasR was required for antibiotic production, pigment biosynthesis, morphological development, chitin and *N*-acetylglucosamine utilization [24]; SACE_Lrp is an efficient regulator for transporting and catabolizing branched-chain amino acids [6]; TFRs were involved in morphological differentiation, propionyl coenzyme A assimilation and erythromycin production [12, 15–17, 25, 26]. Combined with transcriptome data, promoter regions of these transcriptional regulators were selected by EMSAs (Additional file 1: Table S3), and results showed that these transcriptional regulators were not directly regulated by SACE_5754. The direct regulators of *ery* cluster would be exploring in our studies, for a better understanding of regulatory network of erythromycin production.

Manipulating the regulators and their targets is an efficient way to improve antibiotic production in actinomycetes. In an early study, deletion of the Lrp family transcriptional regulator SACE_Lrp in industrial strain WB enhanced the erythromycin production by 19%, while overexpression of its target gene *SACE_5387–5386* in deletion of *SACE_Lrp* of WB increased the Er-A production by 48% [6]. In this study, deletion of *SACE_5754* in the industrial strain WB combined with overexpression of its target genes *SACE_0388* or *SACE_6149* improved Er-A production by 46 and 34%, respectively. When *SACE_0388* and *SACE_6149* were further co-expressed in WBΔ*SACE_5754*, Er-A production was increased by 64% compared to WB.

Our present findings providing a strategy for improved erythromycin production based on engineering of SACE_5754 and its target genes may be applied to the augment of other antibiotic produced by actinomycetes in industry that have SACE_5754 homologs.

## Conclusions

SACE_5754, a TetR family transcriptional regulator, was identified to negatively regulate erythromycin biosynthesis in *S. erythraea*. It indirectly repressed the transcription of *ery* cluster and cannot regulate itself and its adjacent gene. RNA-seq coupled with qRT-PCR, EMSAs and genetic experiments were performed to characterize SACE_5754’s targets associated with erythromycin production. Further engineering of SACE_5754 and its target genes, *SACE_0388* and *SACE_6149*, significantly enhanced erythromycin production relative to its original strain A226. When the combined engineering strategy was carried out in an industrial strain *S. erythraea* WB, erythromycin production was successively increased by 64% in a flask and by 41% in a fermenter. Our work here presents a new knowledge to understand the regulatory mechanism of erythromycin biosynthesis, and provides a useful strategy to identify and dissect target genes of transcriptional factors for improved erythromycin production.

## Methods

### Strains, plasmids, and growth conditions

The strains and plasmids used in this work were listed in Additional file 1: Table S1. *S. erythraea* and its derivatives were grown at 30 °C. Solid R3M medium was used for phenotypic observation and protoplast regeneration [27]. Liquid TSB medium was used for protoplast preparation, seed culture and DNA extraction [28]. The *E. coil* strain DH5α and BL21 (DE3) were cultured in LB liquid medium or on LB solid plates at 37 °C, used to propagate plasmids for routine cloning and as the host for heterologous protein production, respectively [29].

### Gene deletion, complementation, and overexpression

Gene deletion, complementation, and overexpression in *S. erythraea* were showed as previously described [16] . To construct an *SACE_5754* deletion mutant, two 1.5-kb DNA fragments flanking *SACE_5754* were prepared from the genomic DNA of strain *S. erythraea* A226 by PCR using the primer pairs 5754-up-F/R and 5754-down-F/R (Additional file 1: Table S2). The two fragments were digested with *Hin*dIII/*Xba*I and *Kpn*I/*Eco*RI, ligating into the corresponding sites of pUCTSR [25], generating pUCTSR∆*5754*. By the homologous recombination with linearized fragments, a 291-bp fragment of the *SACE_5754* gene was replaced by thiostrepton resistance gene (*tsr*) in *S. erythraea* A226. The desired thiostrepton-resistant mutant, named *∆SACE_5754,* was further confirmed by PCR analysis using the primers 5754-C-F/R (Additional file 1: Table S2).

For complementation of *SACE_5754* in the *∆SACE_5754* and *SACE_5754* overexpression in A226, a 624-bp DNA fragment containing a full-length *SACE_5754* was amplified by PCR with the genomic DNA of A226 as a template. The PCR product was cleaved with *Nde*I/*Xba*I, and inserted into the corresponding sites of integrative expression plasmid pIB139 [30], yielding pIB139–5754. By PEG-mediated protoplast transformation, pIB139–5754 was introduced into the *∆SACE_5754* mutant and the parental strain A226, respectively. The complemented strain *∆SACE_5754/*pIB139–5754 and overexpression strain A226/pIB139–5754 were obtained by apramycin resistance screening and confirmed by PCR analysis with primers Apr-F/R.

In accord with above procedures, we constructed *SACE_0388* deletion mutant *∆SACE_0388*, complemented of *SACE_0388* in the *∆SACE_0388* (*∆SACE_0388/*pIB139–0388), *SACE_0388* overexpression in A226 (A226/pIB139–0388), *SACE_3599* deletion mutant *∆SACE_3599*, *SACE_6149* deletion mutant *∆SACE_6149*, complemented of *SACE_6149* in the *∆SACE_6149* (*∆SACE_6149/*pIB139–6149) and *SACE_6149* overexpression in A226 (A226/pIB139–6149). All primers used in this work were listed in Additional file 1: Table S2.

For *SACE_0388* and *SACE_6149* overexpression in the *∆SACE_5754*, pIB139–0388 and pIB139–6149 were introduced into *∆SACE_5754* strain to obtain *∆SACE_5754/*pIB139–0388 and *∆SACE_5754/*pIB139–6149 by PEG-mediated protoplast transformation, respectively. For co-expression of *SACE_0388* and *SACE_6149* in WB*∆SACE_5754*, the combined DNA fragment containing PermE* and *SACE_6149* from the pIB139–6149 was amplified using the primer pair ermE-F and 6149-C-R. The PCR product was digested with *Not*I/*Eco*RV, ligating into the corresponding sites of pIB1390388 to generate pIB139–0388-6149. By PEG-mediated protoplast transformation, pIB139–0388-6149 was introduced into the WB*∆SACE_5754*. The co-expression *SACE_0388* and *SACE_6149* in the WB*∆SACE_5754* strain WB*∆SACE_5754*/pIB139–0388-6149 was obtained by apramycin resistance screening and confirmed by PCR analysis with primers Apr-F/R.

### Heterologous expression and purification of SACE_5754

The *SACE_5754* coding region of 207 amino acids was obtained by PCR using the primers 5754-22b-F and 5754-22b-R (Additional file 1: Table S2). The PCR production was cut with *Eco*RI/*Hin*dIII and cloned into pET22b generating a C-terminal His-tag fusion. The constructed plasmid pET22b-5754 was introduced into *E. coli* BL21 (DE3), and SACE_5754 expression was induced by 0.5 mM IPTG at 20 °C for18–20 h. The recombinant His_6_-tagged SACE_5754 was extracted and purified on Ni^2+^-NTA spin column (Bio-RAD). The quality of the purified protein was estimated by polyacrylamide gel electrophoresis (SDS-PAGE). The purified protein was stored at 4 °C and used for electrophoretic mobility shift assays (EMSAs) and DNase I footprinting assays.

### Electrophoretic mobility shift assays (EMSAs)

The EMSAs were performed in the light of previously reported methods [31]. DNA probes were amplified by PCR using the primers listed in Additional file 1: Table S2, and were incubated individually with various concentrations of His_6_-tagged SACE_5754 in binding-buffer (10 mM Tris (pH 7.5), 5 mM MgCl_2_, 60 mM KCl, 10 mM DTT, 50 mM EDTA and 10% glycerol) at 30 °C for 10 min in 20 μL reaction mixture. After incubation, the samples were fractionated on 6% native PAGE gels in 1× TAE buffer at 80 mA for 35–45 min.

### DNase I footprinting assay

An FAM fluorescence labeling capillary electrophoresis method was used for DNase I footprinting [32]. To identify the binding site of the SACE_5754 protein in the P_*0388–0389*_ (the entire *SACE_0388*-*SACE_0389* intergenic region), P_*3599*_ (the entire *SACE_3599* intergenic region) and P_*6148–6149*_ (the entire *SACE_6148*-*SACE_6149* intergenic region), a 261-bp 6-carboxyfluorescein (FAM), a 218-bp 6-carboxyfluorescein (FAM) and a 143-bp 6-carboxyfluorescein (FAM) fluorescence-labeled DNA fragment were amplified by PCR using primers FAM-P_*0388–0389*_-F/R, FAM-P_*3599*_-F/R and FAM-P_*6148–6149*_-F/R (Additional file 1: Table S2), respectively. The labeled DNA fragment (150 ng) and corresponding concentrations of His_6_-tagged SACE_5754 protein were added to a reaction mixture (final volume 50 μL), and incubated at 30 °C for 10 min in binding buffer. DNase I (1 U/μg; Promega) treatments with various concentrations were performed for 60 s at 25 °C, and terminated by addition of DNase I Stop Solution and heating for 10 min at 65 °C to inactivate the DNase I. DNA samples were analyzed with a 3730 XL DNA Genetic Analyzer (Applied Biosystems) after purification, and data analyses were performed using GeneMarker software program v2.2.

### RNA isolation and quantitative real-time PCR assay

Total RNA was isolated, using the *TransZol* up (Transgen, China), from cultures of *S. erythraea* A226 and *∆SACE_5754* grown in R5 liquid medium after 2 days. The quality and quantity of RNAs were examined using a microplate reader (BioTek) and confirmed by electrophoresis. The transcription levels of various genes were determined by quantitative real-time PCR analysis as described previously [6] using the primers listed in Additional file 1: Table S2. qRT-PCR was performed on the Applied Biosystems QuantStudio 6 Flex system with Maxima™ SYBR Green/ROX qPCR Master Mix (MBI Fermentas). The *hrdB* (*SACE_1801*) gene was used as the internal control, and relative transcription was quantified using a comparative cycle threshold method [33].

### Fermentation and measurement of erythromycin

For flask fermentation of *S. erythraea* and its derivatives, spores were inoculated into 50 ml of TSB seed medium and grown for 2 days. Then, 5 ml seed culture was transferred into 50 ml R5 liquid medium and all fermentation cultures were grown at 220 rpm, 30 °C for 6 days [27]. For bioreactor cultures, *S. erythraea* WB and its derivatives were cultivated in the industrial medium with a 5-L fermenter (Baoxing, Shanghai, China). Samples (50 ml) were taken every 24 h. Erythromycin was extracted in fermentation culture of the *S. erythraea* strains as described previously [6]. An Agilent Extend-C18 column (5 μm; 250 × 4.6 mm) was equipped in the Waters Breeze HPLC and was equilibrated with 60% solution A (5 mM ammonia acetate, pH 7.0) and 40% solution B (acetonitrile). An isocratic program was performed at a flow rate of 1.0 mL/min at 25 °C using 2424 ELSD detector.

### RNA sequencing

Total RNA was isolated, using the *TransZol* up (Transgen, China), from cultures of *S. erythraea* A226 and *∆SACE_5754* grown in R5 liquid medium after 2 days. The quality and quantity of RNAs were examined using a microplate reader (BioTek) and confirmed by electrophoresis. RNA samples of A226 and *∆SACE_5754* were used for RNA sequencing. Library construction and sequencing were performed using Illumina Hiseq™ 2000 at The Beijing Genomics Institution (BGI, Shenzhen, China).The data obtained from the sequencing of the Illumina HiSeq ™ 2000 is called raw reads or raw data, and then the raw reads were subjected to quality control (QC)-controlled to determine if a resequencing step is needed. After raw reads are filtered, the clean reads were aligned to the reference sequence [34]. The original sequence data have been submitted to the database of NCBI Sequence Read Archive (https://www.ncbi.nlm.nih.gov/sra/SRP129064) under the accession number SRP129064. For gene expression analysis, the matched reads were calculated and then normalized to probability using Noiseq package method [35]. The significance of the differential expression of genes was defined by bioioformatics service of BGI according to the combination of the absolute criteria of Foldchange ≥2 and probability ≥0.8.

To validate the RNA-seq results, 28 genes annotated with biological functions and the probability from high to low were analyzed by qRT-PCR. Our qRT-PCR measurements for these genes showed similar trends of expression changes estimated from the RNA-seq data (Additional file 1: Figure S7).

### Statistical analysis

All data in this study were stated as means ± standard error of mean (SEM), and analysis by Student’s *t*-test, with *p < 0.05, ** *p* < 0.01 and ***p < 0.001, ns, no significant.

## Additional file

Additional file 1: **Table S1.** Strains and plasmids used in this study. **Table S2.** Primers used in this study. **Table S3.** Putative target genes of SACE_5754 were screened by EMSAs. **Figure S1.** Analyses of SACE_5754 homologs and comparison of growth rates and morphological differentiation in *S. erythraea* A226 and relevant strains. **Figure S2.** Inactivation of *SACE_0388* in *S. erythraea* A226. **Figure S3.** Inactivation of *SACE_3599* in *S. erythraea* A226. **Figure S4.** Inactivation of *SACE_6149* in *S. erythraea* A226. **Figure S5.** Effects of *SACE_5754* disruption on transcriptional levels of related genes involving in TCA cycle. **Figure S6.** Possible regulatory pathway of SACE_5754 on erythromycin biosynthesis in *S. erythraea*. **Figure S7.** qRT-PCR analysis of the accuracy of transcriptome analysis. (DOCX 968 kb)

## Abbreviations

6-FAM: 6-isomer of carboxyfluorescein.
CoA: Coenzyme A
EMSA: Electrophoretic mobility shift assay
Er-A: Erythromycin A
*ery*: Erythromycin biosynthetic gene
IPTG: Isopropylthiogalactoside
LB: Luria-Bertani medium
qRT-PCR: Quantitative real-time PCR
SDS-PAGE: Sodium dodecyl sulfate polyacrylamide gel electrophoresis
TCA: Tricarboxylic acid
TFR: TetR family transcriptional regulator
*tsr*: Thiostrepton resistance gene

## References

[1] Butler MS (2008). Natural products to drugs: natural product-derived compounds in clinical trials. Nat Prod Rep.

[2] Barajas JF, Blake-Hedges JM, Bailey CB, Curran S, Keasling JD (2017). Engineered polyketides: synergy between protein and host level engineering. Synth Syst Biotechnol.

[3] Staunton J, Weissman KJ (2001). Polyketide biosynthesis: a millennium review. Nat Prod Rep.

[4] Reeves AR, English RS, Lampel JS, Post DA, Vanden Boom TJ (1999). Transcriptional organization of the erythromycin biosynthetic gene cluster of *Saccharopolyspora erythraea*. J Bacteriol.

[5] Subathra Devi C, Saini A, Rastogi S, Jemimah Naine S, Mohanasrinivasan V (2015). Strain improvement and optimization studies for enhanced production of erythromycin in bagasse based medium using *Saccharopolyspora erythraea* MTCC 1103. 3 Biotech.

[6] Liu J, Chen Y, Wang W, Ren M, Wu P, Wang Y, Li C, Zhang L, Wu H, Weaver DT, Zhang B (2017). Engineering of an Lrp family regulator SACE_Lrp improves erythromycin production in *Saccharopolyspora erythraea*. Metab Eng.

[7] Reeves AR, Brikun IA, Cernota WH, Leach BI, Gonzalez MC, Weber JM (2007). Engineering of the methylmalonyl-CoA metabolite node of *Saccharopolyspora erythraea* for increased erythromycin production. Metab Eng.

[8] van der Heul HU, Bilyk BL, McDowall KJ, Seipke RF, van Wezel GP (2018). Regulation of antibiotic production in Actinobacteria: new perspectives from the post-genomic era. Nat Prod Rep.

[9] Nah JH, Kim HJ, Lee HN, Lee MJ, Choi SS, Kim ES (2013). Identification and biotechnological application of novel regulatory genes involved in *Streptomyces* polyketide overproduction through reverse engineering strategy. Biomed Res Int.

[10] Cuthbertson L, Nodwell JR (2013). The TetR family of regulators. Microbiol Mol Biol Rev.

[11] Ray S, Maitra A, Biswas A, Panjikar S, Mondal J, Anand R (2017). Functional insights into the mode of DNA and ligand binding of the TetR family regulator TylP from *Streptomyces fradiae*. J Biol Chem.

[12] Wu H, Wang Y, Yuan L, Mao Y, Wang W, Zhu L, Wu P, Fu C, Müller R, Zhang L (2016). Inactivation of SACE_3446, a TetR family transcriptional regulator, stimulates erythromycin production in *Saccharopolyspora erythraea*. Synth Syst Biotechnol.

[13] Lei C, Wang J, Liu Y, Liu X, Zhao G, Wang J (2018). A feedback regulatory model for RifQ-mediated repression of rifamycin export in *Amycolatopsis mediterranei*. Microb Cell Factories.

[14] Xu Y, Ke M, Li J, Tang Y, Wang N, Tan G, Wang Y, Liu R, Bai L, Zhang L, et al. TetR-type regulator SLCG_2919 is a negative regulator of lincomycin biosynthesis in *Streptomyces lincolnensis*. Appl Environ Microbiol. 2018;85(1). Print 2019 Jan 1. 10.1128/AEM.02091-18 PMC6293104 30341075

[15] Wu P, Pan H, Zhang C, Wu H, Yuan L, Huang X, Zhou Y, Ye BC, Weaver DT, Zhang L, Zhang B (2014). SACE_3986, a TetR family transcriptional regulator, negatively controls erythromycin biosynthesis in *Saccharopolyspora erythraea*. J Ind Microbiol Biotechnol.

[16] Wu H, Chen M, Mao Y, Li W, Liu J, Huang X, Zhou Y, Ye BC, Zhang L, Weaver DT, Zhang B (2014). Dissecting and engineering of the TetR family regulator SACE_7301 for enhanced erythromycin production in *Saccharopolyspora erythraea*. Microb Cell Factories.

[17] Xu Z, Liu Y, Ye BC. PccD regulates branched-chain amino acid degradation and exerts a negative effect on erythromycin production in *Saccharopolyspora erythraea*. Appl Environ Microbiol. 2018;84. 10.1128/AEM.00049-18 PMC5881055 29439982

[18] Ahn SK, Cuthbertson L, Nodwell JR (2012). Genome context as a predictive tool for identifying regulatory targets of the TetR family transcriptional regulators. PLoS One.

[19] Karnicar K, Drobnak I, Petek M, Magdevska V, Horvat J, Vidmar R, Baebler S, Rotter A, Jamnik P, Fujs S (2016). Integrated omics approaches provide strategies for rapid erythromycin yield increase in *Saccharopolyspora erythraea*. Microb Cell Factories.

[20] Xu JY, Xu Z, Zhou Y, Ye BC (2016). Lysine Malonylome may affect the central metabolism and erythromycin biosynthesis pathway in *Saccharopolyspora erythraea*. J Proteome Res.

[21] Borisova SA, Liu HW (2010). Characterization of glycosyltransferase DesVII and its auxiliary partner protein DesVIII in the methymycin/picromycin biosynthetic pathway. Biochemistry.

[22] Zhang B, Tian W, Wang S, Yan X, Jia X, Pierens GK, Chen W, Ma H, Deng Z, Qu X (2017). Activation of natural products biosynthetic pathways via a protein modification level regulation. ACS Chem Biol.

[23] van Berkel WJ, Kamerbeek NM, Fraaije MW (2006). Flavoprotein monooxygenases, a diverse class of oxidative biocatalysts. J Biotechnol.

[24] Liao CH, Xu Y, Rigali S, Ye BC (2015). DasR is a pleiotropic regulator required for antibiotic production, pigment biosynthesis, and morphological development in *Saccharopolyspora erythraea*. Appl Microbiol Biotechnol.

[25] Han S, Song P, Ren T, Huang X, Cao C, Zhang B (2011). Identification of SACE_7040, a member of TetR family related to the morphological differentiation of *Saccharopolyspora erythraea*. Curr Microbiol.

[26] Yin X, Xu X, Wu H, Yuan L, Huang X, Zhang B (2013). SACE_0012, a TetR-family transcriptional regulator, affects the morphogenesis of *Saccharopolyspora erythraea*. Curr Microbiol.

[27] Kieser T, Buttner M, Chater K, DA H. Practical Streptomyces Genetics. Norwich: The John Innes Foundation; 2000.

[28] Summers RG, Donadio S, Staver MJ, Wendt-Pienkowski E, Hutchinson CR, Katz L (1997). Sequencing and mutagenesis of genes from the erythromycin biosynthetic gene cluster of *Saccharopolyspora erythraea* that are involved in L-mycarose and D-desosamine production. Microbiology.

[29] Sambrook J. RussellDW: Molecular cloning: a laboratory manual. 3rd ed. New York: Cold Spring Harbor Laboratory; 2001.

[30] Wu H, Mao Y, Chen M, Pan H, Huang X, Ren M, Wu H, Li J, Xu Z, Yuan H (2015). Capturing the target genes of BldD in *Saccharopolyspora erythraea* using improved genomic SELEX method. Appl Microbiol Biotechnol.

[31] Hellman LM, Fried MG (2007). Electrophoretic mobility shift assay (EMSA) for detecting protein-nucleic acid interactions. Nat Protoc.

[32] Zianni M, Tessanne K, Merighi M, Laguna R, Tabita FR (2006). Identification of the DNA bases of a DNase I footprint by the use of dye primer sequencing on an automated capillary DNA analysis instrument. J Biomol Tech.

[33] Livak K, Schmittgenb T (2001). Analysis of relative gene expression data using real-time quantitative PCR and the 2^−ΔΔCT^. Methods.

[34] Oliynyk M, Samborskyy M, Lester JB, Mironenko T, Scott N, Dickens S, Haydock SF, Leadlay PF (2007). Complete genome sequence of the erythromycin-producing bacterium *Saccharopolyspora erythraea* NRRL23338. Nat Biotechnol.

[35] Tarazona S, Garcia-Alcalde F, Dopazo J, Ferrer A, Conesa A (2011). Differential expression in RNA-seq: a matter of depth. Genome Res.

