# Supplementary material for: Transcriptome-guided target identification of the TetR-like regulator SACE_5754 and engineered overproduction of erythromycin in Saccharopolyspora erythraea
Source: J Biol Eng. 2019 Jan 24;13:11. doi: 10.1186/s13036-018-0135-2 (PMC6346578; doi:10.1186/s13036-018-0135-2)
Supplement: Supplementary file 1 — Table S1. Strains and plasmids used in this study. Table S2. Primers used in this study. Table S3. Putative target genes of SACE_5754 were screened by EMSAs. Figure S1. Analyses of SACE_5754 homologs and comparison of growth rates and morphological differentiation in S. erythraea A226 and relevant strains. Figure S2. Inactivation of SACE_0388 in S. erythraea A226. Figure S3. Inactivation of SACE_3599 in S. erythraea A226. Figure S4. Inactivation of SACE_6149 in S. erythraea A226. Figure S5. Effects of SACE_5754 disruption on transcriptional levels of related genes involving in TCA cycle. Figure S6. Possible regulatory pathway of SACE_5754 on erythromycin biosynthesis in S. erythraea. Figure S7. qRT-PCR analysis of the accuracy of transcriptome analysis. (DOCX 968 kb) [file 13036_2018_135_MOESM1_ESM.docx]

**Journal of Biological Engineering**

**Additional file 1**

**Transcriptome-guided target identification of the TetR-like regulator SACE_5754 and engineered overproduction of erythromycin in *Saccharopolyspora erythraea***

Hang Wu^1†^, Zuling Chu^1†^, Wanxiang Zhang^1^, Chi Zhang^1^, Jingshu Ni^1^, Heshi Fang^1^, Yuhong Chen^1^, Yansheng Wang^1^, Lixin Zhang^1,2*^, Buchang Zhang^1*^

^1^ School of Life Sciences, Institute of Physical Science and Information Technology, Anhui University, Hefei, 230601, China.

^2^ State Key Laboratory of Bioreactor Engineering, East China University of Science and Technology, Shanghai, 200237, China

^†^ The two authors contributed equally to this work.

* Correspondences:

Buchang Zhang: zhbc@ahu.edu.cn;

Lixin Zhang: lxzhang@ecust.edu.cn

**Table S1 Strains and plasmids used in this study**

| **Strain or plasmid** | **Description** | **Source or reference** |
| --- | --- | --- |
| **Strain** |  |  |
| ***S. erythraea*** |  |  |
| A226 | an erythromycin low producer | [1] |
| ΔbldD | A226 with bldD deleted | [2] |
| ΔSACE_5754 | A226 with SACE_5754 deleted | This study |
| ΔSACE_5754/pIB139 | ΔSACE_5754 carrying pIB139 | This study |
| ΔSACE_5754/pIB139-5754 | ΔSACE_5754 carrying pIB1395754 | This study |
| A226/pIB139 | A226 carrying pIB139 | This study |
| A226/pIB139-5754 | A226 carrying pIB139-5754 | This study |
| ΔSACE_5754/pIB139-0388 | ΔSACE_5754 carrying pIB139-0388 | This study |
| ΔSACE_5754/pIB139-6149 | ΔSACE_5754 carrying pIB139-6149 | This study |
| ΔSACE_0388 | A226 with SACE_0388 deleted | This study |
| ΔSACE_0388/pIB139 | ΔSACE_0388 carrying pIB139 | This study |
| ΔSACE_0388/pIB139-0388 | ΔSACE_0388 carrying pIB139-0388 | This study |
| A226/pIB139-0388 | A226 carrying pIB139-0388 | This study |
| ΔSACE_3599 | A226 with SACE_3599 deleted | This study |
| ΔSACE_6149 | A226 with SACE_6149 deleted | This study |
| ΔSACE_6149/pIB139 | ΔSACE_6149 carrying pIB139 | This study |
| ΔSACE_6149/pIB139-6149 | ΔSACE_6149 carrying pIB139-6149 | This study |
| A226/pIB139-6149 | A226 carrying pIB139-6149 | This study |
| WB | an erythromycin industrial overproducer | [1] |
| WBΔSACE_5754 | WB with SACE_5754 deleted | This study |
| WBΔSACE_5754/pIB139-0388 | WB/ΔSACE_5754 carrying pIB139-0388 | This study |
| WBΔSACE_5754/pIB139-6149 | WB/ΔSACE_5754 carrying pIB139-6149 | This study |
| WBΔSACE_5754/pIB139-0388-6149 | WB/ΔSACE_5754 carrying pIB139-0388-6149 | This study |
| ***E. coil*** |  |  |
| DH5α | F recA lacZM15 | Invitrogen |
| BL21(DE3) | F-ompThsdSB (rB^-^mB^-^) gal dcm (DE3) | Novagen |
| Plasmids |  |  |
| pUCTSR | pUC18 derivative containing a  1.36-kb fragment of a thiostrepton resistance cassette in the *Bam*HI/*Sma*I sites | [3] |
| pUCTSRΔ5754 | pUCTSR derivative for *SACE_5754* deletion | This study |
| pUCTSRΔ0388 | pUCTSR derivative for *SACE_0388* deletion | This study |
| pUCTSRΔ3599 | pUCTSR derivative for *SACE_3599* deletion | This study |
| pUCTSRΔ6149 | pUCTSR derivative for *SACE_6149* deletion | This study |
| pIB139 | *aac*(3)IV, P*ermE** | [4] |
| pIB139-5754 | pIB139 derivative for expression of *SACE_5754* | This study |
| pIB139-0388 | pIB139 derivative for expression of *SACE_0388* | This study |
| pIB139-6149 | pIB139 derivative for expression of *SACE_6149* | This study |
| pIB139-0388-6149 | pIB139 derivative for co-expression of *SACE_0388* and *SACE_6149* | This study |
| pET22b | *kan*, P***_T7_***, His-tag | Novagen |
| pET22b-5754 | pET22b derivative carrying *SACE_5754* | This study |

**Table S2 Primers used in this study**

| **Name** | Sequence (5′-3′) (restriction site underlined) | Use |
| --- | --- | --- |
| 5754-up-F | CCCAAGCTTAGCTCCTCGATCAGCTCCAGG (*Hin*dIII) | Deletion of *SACE_5754* gene |
| 5754-up-R | CTAGTCTAGAGTCGTAGCCCTGCTCGATGA (*Xba*I) |  |
| 5754-down-F | CGGGGTACCGTGATGTTCGGCGGCCTGGTC (*Kpn*I) |  |
| 5754-down-R | CCGGAATTCCCCTGGATGTATGGCCGTCGC (*Eco*RI) |  |
| 5754-C-F | AAATCTAGACACCGCTCACACCGCCATCCTCGCT (*Nde*I) | Confirmation of *SACE_5754* deletion mutant Δ*SACE_5754*, and complementation and overexpression of *SACE_5754* |
| 5754-C-R | CTAGTCTAGACACCGCTCACACCGCCATCCTCGCT (*Xba*I) |  |
| Apr-F | ggAgTgCATATggTgCAATACgAATggCgAAAAg | Confirmation of overexpressing |
| Apr-R | CTCAAAgCTTCAgCCAATCgACTggCgAgCg | strain by PCR |
| 0388-up-F | AACTGCAGCATCCCCGCGAACAGGCCGC (*Pst*I) | Deletion of *SACE_0388* gene |
| 0388-up-R | CTAGTCTAGAGAAGCCAGGCGGTACGCGCA (*Xba*I) |  |
| 0388-down-F | CGGGGTACCCTGTTCCTCACCGCGGGCGG (*Kpn*I) |  |
| 0388-down-R | CCGGAATTCTCTGGCGGGCCACGGACGTT (*Eco*RI) |  |
| 0388-C-F | CGCCATATGATGGCACCCACCGGAGGGCG (*Nde*I) | Confirmation of *SACE_0388* deletion in Δ*SACE_0388*, and complementation and overexpression of *SACE_0388* |
| 0388-C-R | CTAGTCTAGATCAGGTCGCGGCGCTCTCC (*Xba*I) |  |
| 3599-up-F | CCCAAGCTTGGGCAGATCAGGCTGTCAGG (*Hin*dIII) | Deletion of *SACE_3599* gene |
| 3599-up-R | CTAGTCTAGAGGGCATGCGGAAGGGCACGT (*Xba*I) |  |
| 3599-down-F | CGGGGTACCCTGGGCCTGGGCCGGCGGCT (*Kpn*I) |  |
| 3599-down-R | CGAGCTCGTAGAGCAGCAGGCCCATCA (*Sac*I) |  |
| 3599-C-F | CGCCATATG ATGACCAAGCACTTCGCGTT (*Nde*I) | Confirmation of *SACE_3599* |
| 3599-C-R | CTAGTCTAGA TCAGGCCAGGTAGGACTCCA (*Xba*I) | Deletion in Δ*SACE_3599* |
| 6149-up-F | CCCAAGCTTCTCGGTCGGCTCGGCCGGGA (*Hin*dIII) | Deletion of *SACE_6149* gene |
| 6149-up-R | CTAGTCTAGAGCGTCCGGAAGTCGCCGAGC (*Xba*I) |  |
| 6149-down-F | CGGGGTACCCGCCGAACCGCTGCGCGAGA (*Kpn*I) |  |
| 6149-down-R | CCGGAATTC TGGCCGACTACAAGGTCCGC (*Eco*RI) |  |
| 6149-C-F | CGCCATATGATGGAGCGAACGACGTGTTG (*Nde*I) | Confirmation of *SACE_6149* deletion in Δ*SACE_6149*, |
| 6149-C-R | CTAGTCTAGATCAGCGCAGCGACACCTTCG (*Xba*I) | and complementation and overexpression of *SACE_6149* |
| ermE-F  6149-F | ATTTGCGGCCGCGCGAGTGTCCGTTCGAGTGGCG (*Not*I)  AAAAGATATCTCAGCGCAGCGACACCTTCGGC (*Eco*RV) | Cloning of combined DNA fragment containing *PermE** and *SACE_6149* |
| 5754-22b-F | CCGGAATTCGTGAGCGGTGGCATCGAGGA (*Eco*RI) | Expression of SACE_5754 in *E. coli* |
| 5754-22b-R | CCCAAGCTTCGACGGCACGTCCGACAGGT (*Hin*dIII) |  |
| P5753-5754-F | GACTGATCTTGCCGATGTCG | Probe *SACE_5753-5754*-int |
| P5753-5754-R | TCGGCGCCTCCTCCTCGATG |  |
| P0388-0389-F  P0388-0389-R | TGCCCGAAAGGTAACCCCGC  CCCCCGGCGAGCGCAACCGA | Probe *SACE_0388-*0389-int |
| P3599-F  P3599-R | TCACCACCTGAAACCGCCAC  TGCTTGGTCATGGGTTCCTC | Probe SACE_3599-int |
| P6148-6149-F  P6148-6149-R | GGTGGCTTCTGGCGTGCTCA  GCAACACGTCGTTCGCTCCA | Probe *SACE_6148-*6149-int |
| P*eryC*I-*ermE-*F | GTGCTGCCGATCCTGGTTGCG | Probe *eryC*I-*ermE*-int |
| P*eryC*I-*ermE-*R | CGGACCGGAGTTCGAGGTACGC |  |
| P*eryB*I-*B*III-F  PeryBI-BIII-R | GCAGCACGGACTCGAGGTCATGG  GATACGGCCGCCACGAGCAGC | Probe *eryB*I-*B*III-int |
| P*eryA*I-*B*IV-F | CCGCGCTCCCGACGAAGCC | Probe *eryA*I-*B*IV-int |
| P*eryA*I-*B*IV-R | CACGGACGAACGATCCTCCCAGG |  |
| Pery*BV*I-F | GCCGAGCAGGAAACGCGTTGC | Probe ery*BV*I-int |
| Pery*BV*I-R | CTGTGCAGTACCTGGAGGGCGGAC |  |
| P*eryK-F*  P*eryK*-R | GGACCGGATCCGCTCCCAGAG  CATCGTGCCCAGCCAGTCGAGG | Probe *eryK*-int |
| FAM- P0388-0389-F  P0388-0389-R | TGCCCGAAAGGTAACCCCGC  CCCCCGGCGAGCGCAACCGA | Probe *SACE_0388-*0389-int |
| FAM-P3599-F  P3599-R | TCACCACCTGAAACCGCCAC  TGCTTGGTCATGGGTTCCTC | Probe SACE_3599-int |
| FAM-P6148-6149-F  P6148-6149-R | GGTGGCTTCTGGCGTGCTCA  GCAACACGTCGTTCGCTCCA | Probe *SACE_6148-*6149-int |
| Probe1-F  Probe1-R | GGCGGGCAGGAAATCAACACTTGTTGAATTACGGTCGCGCA  CCCCCGGCGAGCGCAACCGATTTGATTGCAGCAACCAACT | Probe 1 |
| Probe2-F  Probe2-R | GGCGGGCAGGCCCTCAACAAGTGTTGACGGCCGGTCGCGCA  CCCCCGGCGAGCGCAACCGATTTGATTGCAGCAACCAACT | Probe 2 |
| Probe3-F  Probe3-R | GGCGGGCAGGAAAGACCACCTGTGGTCATTACGGTCGCGCA  CCCCCGGCGAGCGCAACCGATTTGATTGCAGCAACCAACT | Probe 3 |
| Probe4-F  Probe4-R | ACCCGCACGCACGTTGAATTCAACACATGTTCAACAGCGTCGGAGGCG  TGCTTGGTCATGGGTTCCTCACGCGGACGATGGCGGCCGT | Probe 4 |
| Probe5-F  Probe5-R | ACCCGCACGCAAGTTGAAGGACCACACGTTTCAACC GCGTCGGAGGCG  TGCTTGGTCATGGGTTCCTCACGCGGACGATGGCGGCCGT | Probe 5 |
| Probe6-F  Probe6-R | ACCCGCACGCACTGGTCCTTCAACACATGGGACCAA GCGTCGGAGGCG  TGCTTGGTCATGGGTTCCTCACGCGGACGATGGCGGCCGT | Probe 6 |
| Probe7-F  Probe7-R | GGTGGCTTCTGGCGTGCTCATAACGCGTTCAGCGTATCC  CGATCCTCCAAGTTTTCAACGCATGTTGAATAACTTCAGGGTGC | Probe 7 |
| Probe8-F  Probe8-R | GGTGGCTTCTGGCGTGCTCATAACGCGTTCAGCGTATCC  CGATCCTCCACTGGTTCAACTACGGTTGAAGCCATTCAGGGTGC | Probe 8 |
| Probe9-F  Probe9-R | GGTGGCTTCTGGCGTGCTCATAACGCGTTCAGCGTATCC  CGATCCTCCAAGTTGGACCAGCATTGGTCCTAACTTCAGGGTGC | Probe 9 |
| hrdB-RT-F  hrdB-RT-R | CGATACGGGCAGACTTGA  CGTTTACGGCGTGGACTA | qRT-PCR analysis of *hrdB*  as an internal control |
| eryK-RT-F  eryK-RT-R | GAAACCGCACTCCTCGACT  GTCGGGTCGGAGGAGAAC | qRT-PCR analysis of *eryK* |
| eryBVI-RT-F  eryBVI-RT-R | GGTCCGGTTCATCGAGTACT  AACACCTCGACGACCATGTT | qRT-PCR analysis of *eryBVI* |
| eryBIV-RT-F  eryBIV-RT-R | CTGCTGCACGTCGAGGAC  GAGACGGCCCGGAAGATG | qRT-PCR analysis of *eryBIV* |
| eryAI-RT-F  eryAI-RT-R | CAAGATGGTGCTGGCGATGC  GCTGATGCCGAACGACGAGAC | qRT-PCR analysis of *eryAI* |
| eryBIII-RT-F  eryBIII-RT-R | CTACCTGCCCTCCATGCTC  GTAGACGTCGGTGATCTCGG | qRT-PCR analysis of *eryBIII* |
| eryBI-RT-F  eryBI-RT-R | AACTTCGAGACCTTCAGCGA  GTCGGTCTCCTGGGTGTTG | qRT-PCR analysis of *eryBI* |
| ermE-RT-F  ermE-RT-F | GTCAACGCCGACTTCCTCACC  CTCCAGGCACCAGTCCACGAT | qRT-PCR analysis of *ermE* |
| eryCI-RT-F  eryCI-RT-R | CGGAACTGAAGGACGTCC  GAGGACGAAGAGGTGCCAG | qRT-PCR analysis of *eryCI* |
| 5753-RT-F  5753-RT-R | GGAGAAGGAGCTCATCAACG  AGAAGTCCACGACCTTCACC | qRT-PCR analysis of *SACE_5753* |
| 5754-RT-F  5754-RT-R | GGCATGGTCCGCTACGTC  GGTCAGGTAGCGCTGGAG | qRT-PCR analysis of *SACE_5754* |
| 0388-RT-F | CACAACGTCACCACCGAGA | qRT-PCR analysis of *SACE_0388* |
| 0388-RT-R | CGGTCGAAGAACGACTCCA |  |
| 3599-RT-F | GATGCGGTCTGCTACGACA | qRT-PCR analysis of *SACE_3599* |
| 3599-RT-R | CGTTTCGTTGGAGGCGAAG |  |
| 6149-RT-F | ATCATCGCCAAAGACACCGA | qRT-PCR analysis of *SACE_6149* |
| 6149-RT-R | GTGACGTGCAGGAGCTTCA |  |
| 0150-RT-F  0150-RT-R | GTTCGGACGTGGTGATCAAG  CTGGTCTTCTCGGTGCGG | qRT-PCR analysis of *SACE_0150* |
| 0401-RT-F  0401-RT-R | CATCTCGCACTGCTGGAAC  GACATCTTGGCCAGCTTGTG | qRT-PCR analysis  of *SACE_0401* |
| 1037-RT-F | AACCTGTTCCTGGACATGGC | qRT-PCR analysis of *SACE_1037* |
| 1037-RT-R | AGGTGGGTGTCGTTGAACA |  |
| 1242-RT-F | ACCAAACACCACATCCCGAT | qRT-PCR analysis of *SACE_1242* |
| 1242-RT-R | TCGGTGCATGATCTTCTCGG |  |
| 1243-RT-F | ATCAACTCGATGGGCTTCCG | qRT-PCR analysis of *SACE_1243* |
| 1243-RT-R | AGAACTCGCAGGACACGATG |  |
| 1833-RT-F | GTATGCCGATCCCAGCGT | qRT-PCR analysis of *SACE_1833* |
| 1833-RT-R | CCTCGCCGATCTCCTTGTT |  |
| 1895-RT-F | CAGACACCCGACTACATCCG | qRT-PCR analysis of *SACE_1895* |
| 1895-RT-R | CCGAGAAGAGGGGTGGATTG |  |
| 1912-RT-F | CAAGGCGGTCAACATCGTG | qRT-PCR analysis of *SACE_1912* |
| 1912-RT-R | TTCTTGGCGTTCTTGGTGAC |  |
| 2271-RT-F | GTCAAGGTGCTGGCCAAC | qRT-PCR analysis of *SACE_2271* |
| 2271-RT-R | CCAGGAAGCAGAACTCGGT |  |
| 2470-RT-F | CAGATCATCGTCGCGCAG | qRT-PCR analysis of *SACE_2470* |
| 2470-RT-R | CAAGGGCAGTGCAGTTCG |  |
| 2475-RT-F | CAACATCACCAACCCGAACA | qRT-PCR analysis of *SACE_2475* |
| 2475-RT-R | GTCGAAGTAGGCATCCAGGT |  |
| 3025-RT-F | CAACCTCGGTATCCCCAAGT | qRT-PCR analysis |
| 3025-RT-R | CCCGAAGCTGGTCATGGC | of *SACE_3025* |
| 3061-RT-F  3061-RT-R | CTTCCCGGACAAGTTCATGC  GACGTTGCGATTGGTGAAAC | qRT-PCR analysis of *SACE_3061* |
| 3062-RT-F  3062-RT-R | GAGCACCTCCTCGATCTTCC  CCATGTTCTGCGCGTAGATC | qRT-PCR analysis of *SACE_3062* |
| 3770-RT-F  3770-RT-F | GTGATGACCTGGCGCATTTG CCAACTCCTCGAGCAACCAA | qRT-PCR analysis of *SACE_3770* |
| 3888-RT-F  3888-RT-R  3897-RT-F  3897-RT-R | ATTCCTTCCTCACCGCCTAC  CTTGCGGACGGTTACCAGT  AGGTCAAGACGATCGAGGAC  TGCTTCATCTTCGCCATGTG | qRT-PCR analysis of *SACE_3888*  qRT-PCR analysis of *SACE_3897* |
| 3901-RT-F  3901-RT-R | CAAGTACACGTTCCCGGACA  CTGGAAATCGCCGTAGGTGT | qRT-PCR analysis of *SACE_3901* |
| 3996-RT-F  3996-RT-R | GTCACCCGGATCGAGACC  GGGAACTCGAACGGGATCA | qRT-PCR analysis of *SACE_3996* |
| 4025-RT-F  4025-RT-R | CAGGAGTCACACCCGAAGTC  GGAACGAGATCCTTCGCGTC | qRT-PCR analysis of *SACE_4025* |
| 4028-RT-F  4028-RT-R | GCTGTGGATCATCGACTGGA  GGCCTGGATCTGCTGGTAG | qRT-PCR analysis of *SACE_4028* |
| 6150-RT-F  6150-RT-R | GGTCTCCCGGATCATGACG  ATGGAGACCGAGACCTTCTG | qRT-PCR analysis of *SACE_6150* |
| 6340-RT-F  6340-RT-R | CTACGTGCTGACCAACAACC  GACCTGGTCGAGCTTGATCA | qRT-PCR analysis of *SACE_6340* |
| 7047-RT-F  7047-RT-R | CTGTACGTGTTCCTGACCCT  CCTGGATGCTGGGGATCG | qRT-PCR analysis of *SACE_7047* |
| 0649-RT-F  0649-RT-R | GGACCTCAAGCAGTTCTTCG  GGCTGTCCTGGTAGAAGGTG | qRT-PCR analysis of *SACE_0649* |
| 1171-RT-F  1171-RT-R | GTGCAACATCACCAAGTGCT  CCTCAGGATGGGGTCGTAG | qRT-PCR analysis of *SACE_1171* |
| 3674-RT-F  3674-RT-R | GAAGGCCTGATCTCCTCCTT  CTCTTCGCTGAGCTCGTTG | qRT-PCR analysis of *SACE_3674* |
| 5638-RT-F  5638-RT-R | AACAGGTGGAGAAGGTCCTG  ACGGTGATGCCGTCGTAG | qRT-PCR analysis of *SACE_5638* |
| 5639-RT-F  5639-RT-R | GGACCATCCAGAACGACATC  GTAGGCGAAGATGTCGGAGA | qRT-PCR analysis of *SACE_5639* |
| 6668-RT-F  6668-RT-R | CTTCCTCAACGACAACAGCA  GTTGACACCACCCACGATGT | qRT-PCR analysis of *SACE_6668* |
| 6669-RT-F  6669-RT-R | GTACCAGGCGAAGGAACTCTT  GTCTTCACCTGGGCTTTGAC | qRT-PCR analysis of *SACE_6669* |
| 7274-RT-F  7274-RT-R | TCAAACGGCTGGAGAAGAAG  GGCTGCATCCAGTAGTTGG | qRT-PCR analysis of *SACE_7274* |

**Table S3 Putative target genes of SACE_5754 were screened by EMSAs**

| **Gene ID** | **Log2^(Δ^*^SACE_5754^*^/A226)^** | **Probability** | **Function** |
| --- | --- | --- | --- |
| SACE_4863 | 13.70628016 | 0.961058 | hypothetical protein |
| SACE_0388 | 8.976779359 | 0.953873 | pyruvate, water dikinase |
| SACE_3068 | -13.47661965 | 0.953801 | hypothetical protein |
| SACE_3888 | 12.79075575 | 0.923409 | CorA-like putative magnesium transport protein |
| SACE_6149 | 6.498149359 | 0.916655 | monooxygenase%2C FAD-binding |
| SACE_3931 | 5.845986323 | 0.914427 | hypothetical protein |
| SACE_0401 | 5.948043092 | 0.913781 | MerR-family transcriptional regulator |
| SACE_6340 | 6.598138279 | 0.913278 | trypsin-like serine protease |
| SACE_1833 | 4.879788544 | 0.913062 | two-component system response regulator |
| SACE_2475 | 4.998822934 | 0.912847 | multicopper oxidase type I |
| SACE_2271 | -4.958348962 | 0.912775 | putative phosphoenolpyruvate-protein phosphotransferase enzyme I |
| SACE_4027 | -4.219426159 | 0.912344 | hypothetical protein |
| SACE_3599 | 5.643747801 | 0.911482 | antibiotic resistance macrolide glycosyltransferase |
| SACE_1242 | -3.884339861 | 0.910979 | cytochrome P450 |
| SACE_1392 | 4.691828889 | 0.910979 | hypothetical protein |
| SACE_2275 | -4.60359549 | 0.910979 | phosphotransferase system fructose IIC component |
| SACE_2274 | -4.68296222 | 0.910835 | putative fructose-specific permease |
| SACE_1912 | 4.755047873 | 0.910763 | putative alkaline serine protease |
| SACE_2470 | 5.003819049 | 0.910404 | iron sulphur binding protein |
| SACE_1243 | -3.749137571 | 0.910116 | putative type III polyketide synthase |
| SACE_3771 | 4.274605023 | 0.910045 | hypothetical protein |
| SACE_3901 | 3.88363731 | 0.909757 | glycine sarcosine N-methyltransferase |
| SACE_0213 | 3.727818337 | 0.909613 | hypothetical protein |
| SACE_1396 | 3.726158416 | 0.909039 | hypothetical protein |
| SACE_0212 | 3.690613087 | 0.908967 | hypothetical protein |
| SACE_1037 | 3.975704143 | 0.908895 | putative ECF family RNA polymerase sigma factor |
| SACE_3062 | -3.687103 | 0.908895 | hydrogen:quinone oxidoreductase |
| SACE_3025 | 4.223383419 | 0.908823 | PE-PGRS family protein |
| SACE_0150 | 3.694035686 | 0.908751 | heat shock protein HSP20 |
| SACE_1241 | -3.605824309 | 0.908679 | hypothetical protein |
| SACE_0372 | 3.871684587 | 0.908608 | hypothetical protein |
| SACE_3996 | 4.521408211 | 0.908392 | putative sporulation-control protein |
| SACE_6150 | 4.34779059 | 0.908105 | 5-oxopent-3-ene-1,2,5-tricarboxylate decarboxylase |
| SACE_3061 | -3.594902182 | 0.907961 | NADH ubiquinone oxidoreductase, 20 kDa subunit |
| SACE_7047 | 3.816640175 | 0.907889 | probable sugar efflux transporter, MFS superfamily |
| SACE_4028 | -4.038695303 | 0.907817 | ABC transporter protein, ATP-binding component |
| SACE_3770 | 4.332639807 | 0.907745 | Asp/Glu racemase |
| SACE_0918 | 3.47739354 | 0.907674 | hypothetical protein |
| SACE_4025 | -4.225600749 | 0.907674 | membrane translocator |
| SACE_1895 | 4.075942135 | 0.907171 | transcriptional regulator, XRE family |
| SACE_6783 | 3.783918955 | 0.907171 | hypothetical protein |
| SACE_2273 | -4.262767087 | 0.906955 | putative 6-phosphofructokinase |
| SACE_0067 | 3.308604466 | 0.906739 | hypothetical protein |
| SACE_3897 | 3.640918711 | 0.906668 | putative S-adenosyl-L-homocysteine hydrolase |
| SACE_3899 | 3.668543039 | 0.906596 | adenosine kinase |
| SACE_2479 | 4.028083682 | 0.906237 | secreted trypsin-like serine protease |
| SACE_3066 | -3.47676695 | 0.905734 | hypothetical protein |
| SACE_2277 | -4.345691301 | 0.90559 | levanase |
| SACE_2471 | 5.538776064 | 0.90559 | hypothetical protein |
| SACE_2010 | 3.665334228 | 0.905518 | putative glycosyltransferase |
| SACE_3898 | 3.7324472 | 0.905518 | putative 5-methyltetrahydrofolate:homocysteine S-methyltransferase |
| SACE_3408 | -4.058304714 | 0.904943 | hypothetical protein |
| SACE_6027 | 4.76525114 | 0.904943 | iron transport protein-binding protein |
| SACE_3063 | -3.356736267 | 0.904871 | nitrogen-fixing NifU-like |
| SACE_3067 | -3.506131635 | 0.904584 | hypothetical protein |
| SACE_0060 | 3.847626237 | 0.904512 | hypothetical protein |
| SACE_3932 | 5.204378929 | 0.904512 | oxidoreductase |
| SACE_1606 | 5.095906633 | 0.904297 | light-dependent protochlorophyllide reductase |
| SACE_1393 | 3.24755403 | 0.904153 | hypothetical protein |
| SACE_3900 | 3.16288945 | 0.904009 | putative S-adenosylmethionine synthetase |
| SACE_2478 | 4.205065604 | 0.903937 | transposase |
| SACE_4862 | 4.740013527 | 0.903937 | Na(+)-linked D-alanine glycine permease |
| SACE_4026 | -4.241719281 | 0.903865 | hypothetical protein |
| SACE_1832 | 3.929855692 | 0.90365 | putative ribonuclease D |
| SACE_5168 | -4.39478809 | 0.90365 | acyl-CoA dehydrogenase |
| SACE_1394 | 3.206503354 | 0.902716 | hypothetical protein |
| SACE_3064 | -3.328873857 | 0.902357 | hypothetical protein |
| SACE_2469 | 5.195735352 | 0.902285 | DsbA oxidoreductase |
| SACE_5747 | -2.97237935 | 0.901854 | hypothetical protein |
| SACE_1295 | 3.498579338 | 0.901566 | hypothetical protein |
| SACE_0068 | 2.993696829 | 0.901494 | hypothetical protein |
| SACE_1053 | -3.010086543 | 0.901494 | hypothetical protein |
| SACE_3060 | -3.109885243 | 0.901063 | hypothetical protein |
| SACE_2276 | -4.930066841 | 0.900992 | phosphocarrier, HPr family |
| SACE_6639 | -3.053402122 | 0.900848 | hypothetical protein |
| SACE_4029 | -3.644171424 | 0.900776 | putative ABC transporter ATP-binding protein |
| SACE_2272 | -3.552212418 | 0.900632 | transcriptional regulator, DeoR family |
| SACE_3065 | -3.098065927 | 0.900345 | hypothetical protein |
| SACE_1240 | -3.310238851 | 0.900201 | integral membrane protein |
| SACE_3385 | 3.806885799 | 0.900057 | clp protease ATP binding subunit |
| SACE_0500 | -0.978878001 | 0.715045265 | putative GntR-family transcriptional regulator |
| SACE_6128 | 1.748325296 | 0.839416583 | putative transcriptional regulator, AsnC family |
| SACE_5717 | -1.576881139 | 0.801911194 | transcriptional regulator, AsnC family |
| SACE_5667 | 2.039537826 | 0.783374048 | AsnC-family transcriptional regulator |
| SACE_5388 | 0.223435345 | 0.115964937 | leucine responsive regulator |
| SACE_7197 | 1.518661 | 0.772237 | TetR-family transcriptional regulator |
| SACE_0646 | 1.506317 | 0.755712 | possible transcriptional regulator, TetR family |
| SACE_7301 | 1.167924 | 0.722805 | transcriptional regulator (TetR/AcrR family) |
| SACE_2191 | 1.04768 | 0.687455 | possible transcriptional regulator, TetR family |
| SACE_3980 | 1.686971 | 0.677468 | TetR-family transcriptional regulator |
| SACE_4216 | -1.17196 | 0.674163 | putative TetR-family transcriptional regulator |
| SACE_4682 | 3.372465 | 0.663026 | transcriptional regulator, TetR family |
| SACE_1996 | 1.122641 | 0.654045 | probable transcriptional regulator, TetR family |
| SACE_3451 | -0.95648 | 0.647507 | transcriptional regulator, TetR family |
| SACE_3428 | -1.00339 | 0.646788 | TetR-family transcriptional regulator |
| SACE_7056 | -1.54584 | 0.644417 | transcriptional regulator, TetR family |
| SACE_5370 | 1.248809 | 0.644417 | TetR-family transcriptional regulator |
| SACE_3302 | -1.34377 | 0.626239 | TetR-family transcriptional regulator |
| SACE_0065 | -0.65249 | 0.62121 | TetR-family transcriptional regulator |
| SACE_1874 | 2.776723 | 0.610864 | TetR-family transcriptional regulator |
| SACE_5390 | 0.740708 | 0.606696 | TetR-family transcriptional regulator |
| SACE_0659 | 0.873161 | 0.604972 | TetR-family transcriptional regulator |
| SACE_1058 | 0.916841 | 0.596925 | transcriptional regulator, TetR family |
| SACE_6360 | 1.025369 | 0.590315 | transcriptional regulator (TetR/AcrR family) |
| SACE_4889 | -0.65609 | 0.587513 | transcriptional regulator, TetR family |
| SACE_7325 | 1.548462 | 0.581046 | transcriptional regulator, TetR family |
| SACE_2879 | 1.562661 | 0.557264 | TetR-family transcriptional regulator |
| SACE_1632 | -0.68442 | 0.534703 | putative TetR-type transcriptional regulator |
| SACE_2679 | -0.85069 | 0.513077 | transcriptional regulator, TetR family |
| SACE_7371 | 1.159673 | 0.505173 | transcriptional regulator, TetR family |
| SACE_6185 | 0.842392 | 0.502227 | transcriptional regulator, TetR family |
| SACE_3446 | 1.054837 | 0.452579 | putative transcriptional regulator of the TetR family |
| SACE_7040 | 0.171024 | 0.181995 | putative TetR family transcriptional regulator |
| SACE_3986 | -0.18917 | 0.138885 | possible transcriptional regulator, TetR family |
| SACE_0012 | -0.07329 | 0.100661 | TetR-family transcriptional regulator |

**Figure S1**


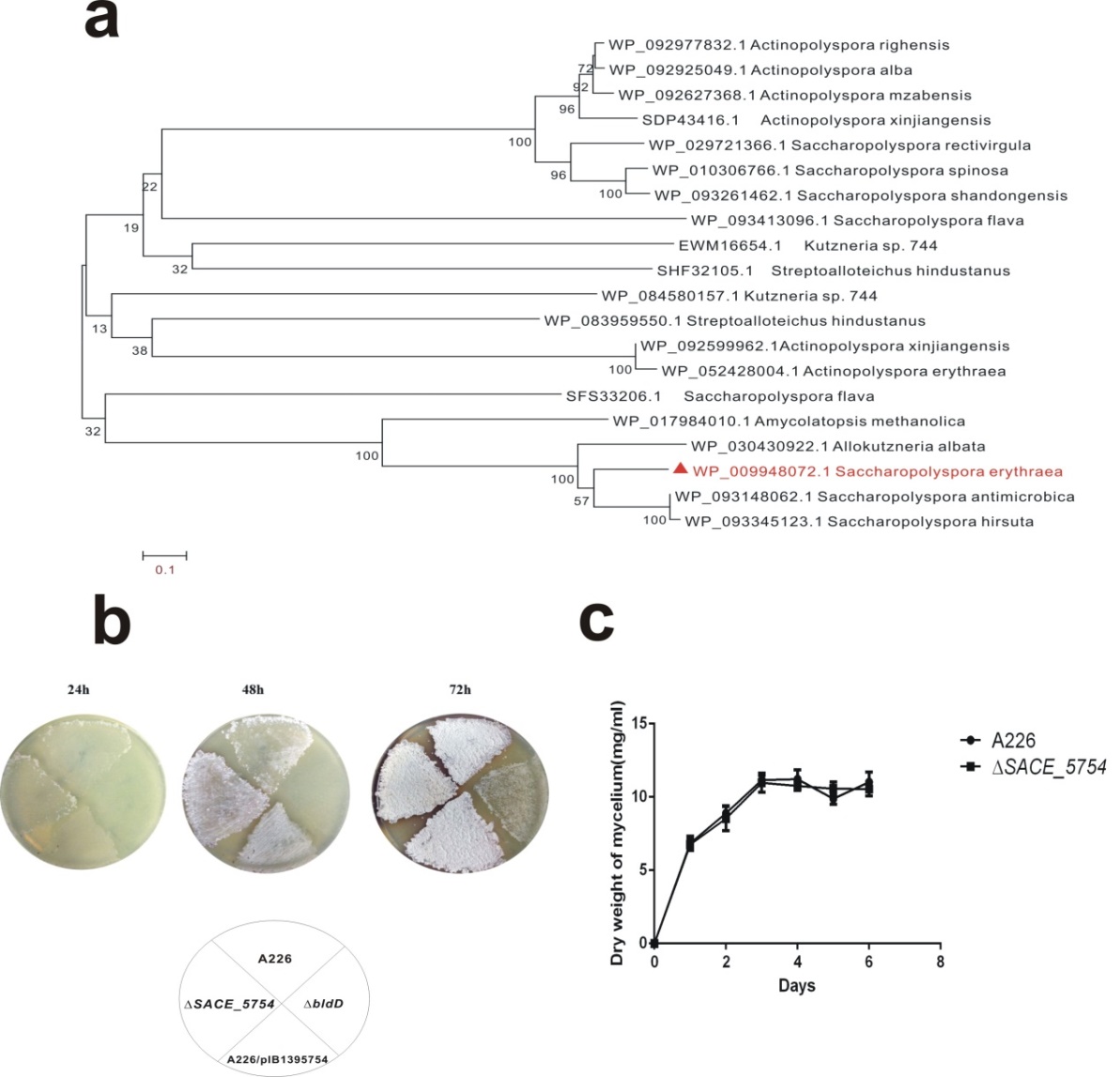


**Figure S1 Analyses of SACE_5754 homologs and Comparison of growth rates and morphological differentiation in *Saccharopolyspora erythraea* A226 and relevant strains.**

**(a)** Neighbor-joining (NJ) distance tree constructed using amino acid sequence of SACE_5754 from the actinomycetes using MEGA6. The NCBI database accession numbers of the sequence used in this analysis come from different actinomycetes species. **(b)** Aerial mycelia formation of A226, ∆*SACE_5754*, A226/pIB139-5754 and ∆*bldD*. All strains were grown on R3M agar medium at 30℃ for 24 h, 48 h and 72 h. **(c)** Growth curves of A226 and ∆*SACE_5754*. The two strains were cultured in the R5 liquid medium, and their dry weights of mycelia (DWM) were measured.

**Figure S2**


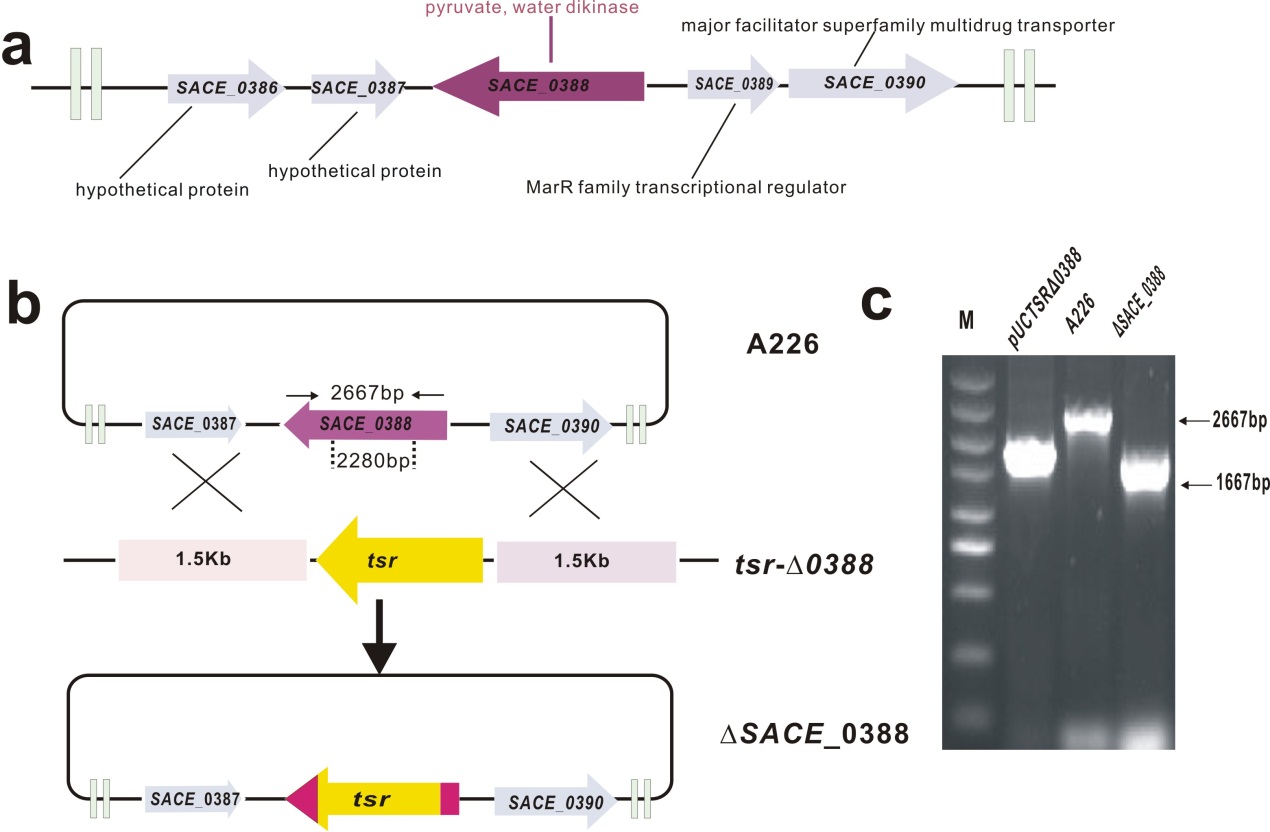


**Figure S2 Inactivation of *SACE_0388* in *S. erythraea* A226.**

**(a)** Genetic organization of *SACE_0388* and its adjacent genes in *S. erythraea*. **(b)** Schematic deletion of *SACE_0388* by linearized fragment homologous recombination in *S. erythraea* A226. **(c)** PCR confirmation of the *SACE_0388* deletion mutant by primers 0388-C-F and 0388-C-R. Lanes: M, 5000-bp DNA ladder; pUCTSR∆*0388*, the positive control, 1667 bp amplified from pUCTSR∆*0388*; A226, the negative control, 2667 bp amplified from A226; ∆*SACE_0388*, 1727 bp amplified from ∆*SACE_0388*.

**Figure S3**

**
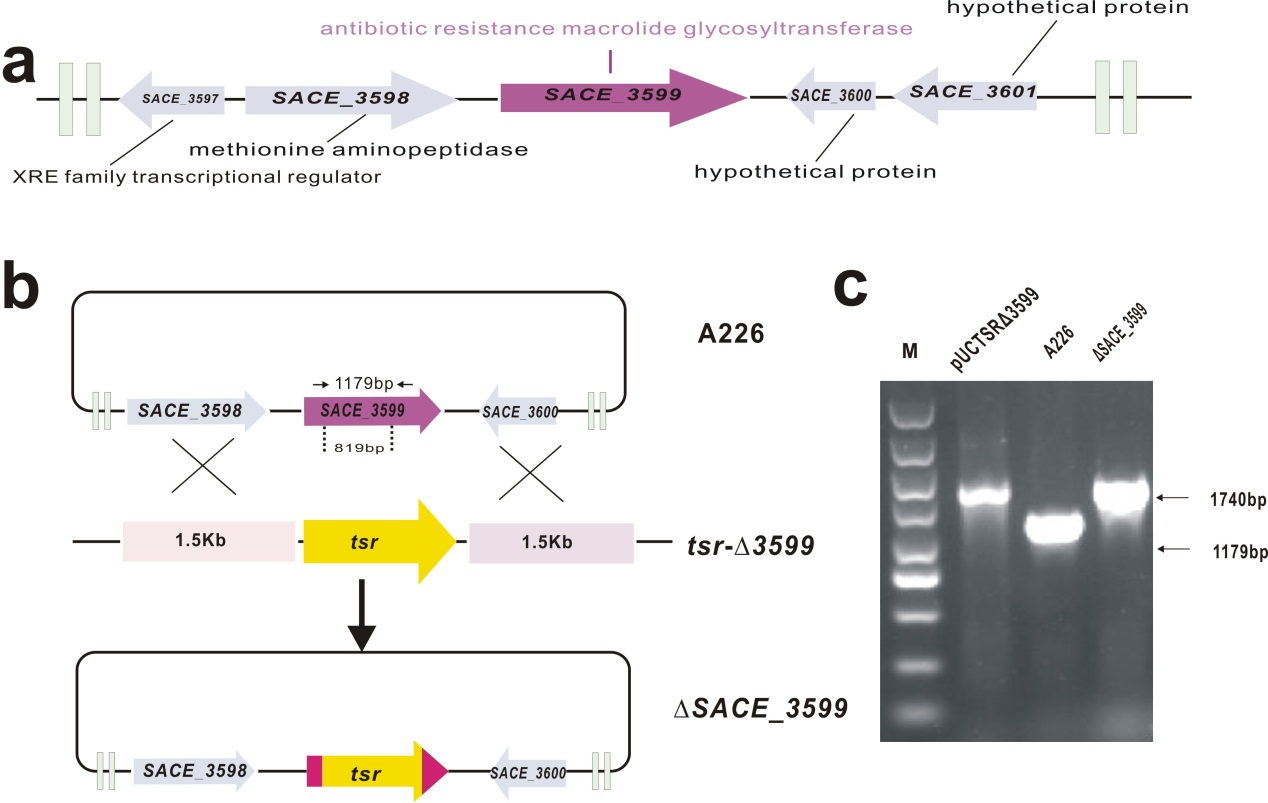
**

**Figure S3 Inactivation of *SACE_3599* in *S. erythraea* A226.**

**(a)** Genetic organization of *SACE_3599* and its adjacent genes in *S. erythraea*. **(b)** Schematic deletion of *SACE_3599* by linearized fragment homologous recombination in *S. erythraea* A226. **(c)** PCR confirmation of the *SACE_3599* deletion mutant by primers 3599-C-F and 3599-C-R. Lanes: M, 5000-bp DNA ladder; pUCTSR∆*3599*, the positive control, 1740 bp amplified from pUCTSR∆*3599*; A226, the negative control, 1179 bp amplified from A226; ∆*SACE_3599*, 1740 bp amplified from ∆*SACE_5754*.

**Figure S4**

**
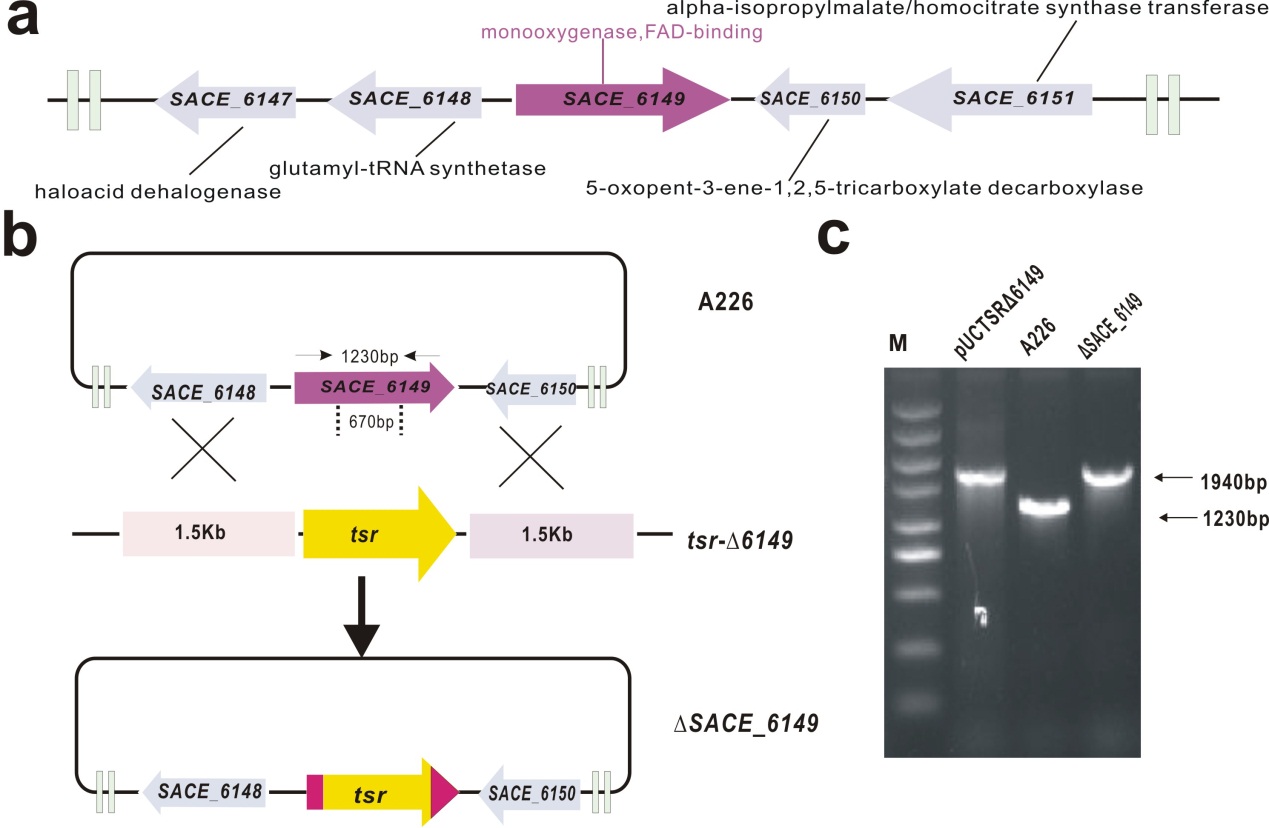
**

**Figure S4 Inactivation of *SACE_6149* in *S. erythraea* A226.**

**(a)** Genetic organization of *SACE_6149* and its adjacent genes in *S. erythraea*. **(b)** Schematic deletion of *SACE_6149* by linearized fragment homologous recombination in *S. erythraea* A226. **(c)** PCR confirmation of the *SACE_6149* deletion mutant by primers 6149-C-F and 6149-C-R. Lanes: M, 5000-bp DNA ladder; pUCTSR∆*6149*, the positive control, 1940 bp amplified from pUCTSR∆*6149*; A226, the negative control, 1230 bp amplified from A226; ∆*SACE_6149*, 1940 bp amplified from ∆*SACE_6149*.

**Figure S5**


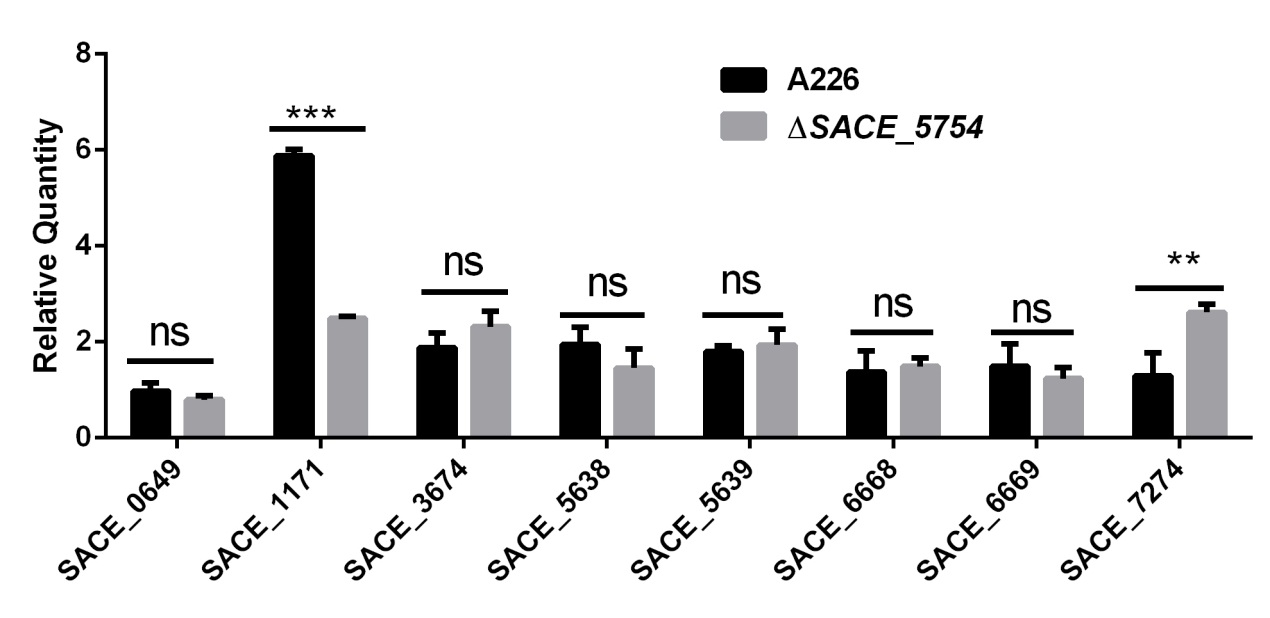


**Figure S5 Effects of *SACE_5754* disruption on transcriptional levels of related genes involving in TCA cycle.** qRT-PCR was used to quantify the amounts of transcripts in A226 and Δ*SACE_5754* cultured for 48 h in liquid R5 medium**.** The Mean values of three replicates are shown, with the standard deviation indicated by error bars. **p* <0.05, ***p* <0.01, ****p* <0.001, ns, no significant.

**Figure S6**


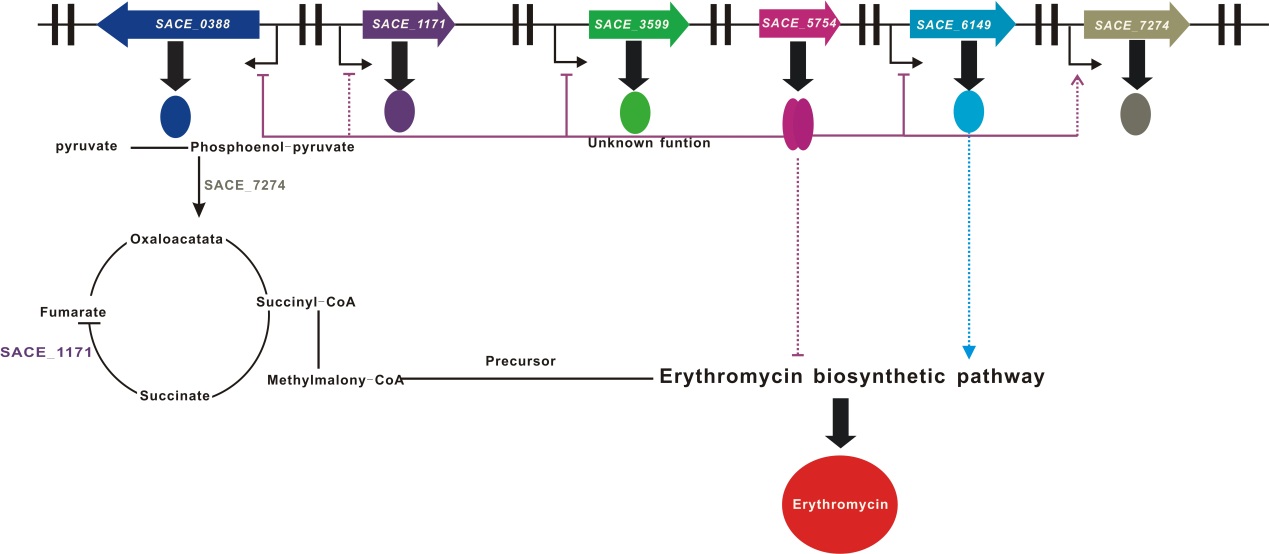


**Figure S6 Possible regulatory pathway of SACE_5754 on erythromycin biosynthesis in *S. erythraea*.** Purple bars, repression; Purple arrows, activation; black bars, repression; black arrows, activation Blue arrows, positive effect on erythromycin biosynthesis; solid lines: direct control; dashed lines: indirect control.

**Figure S7**

**
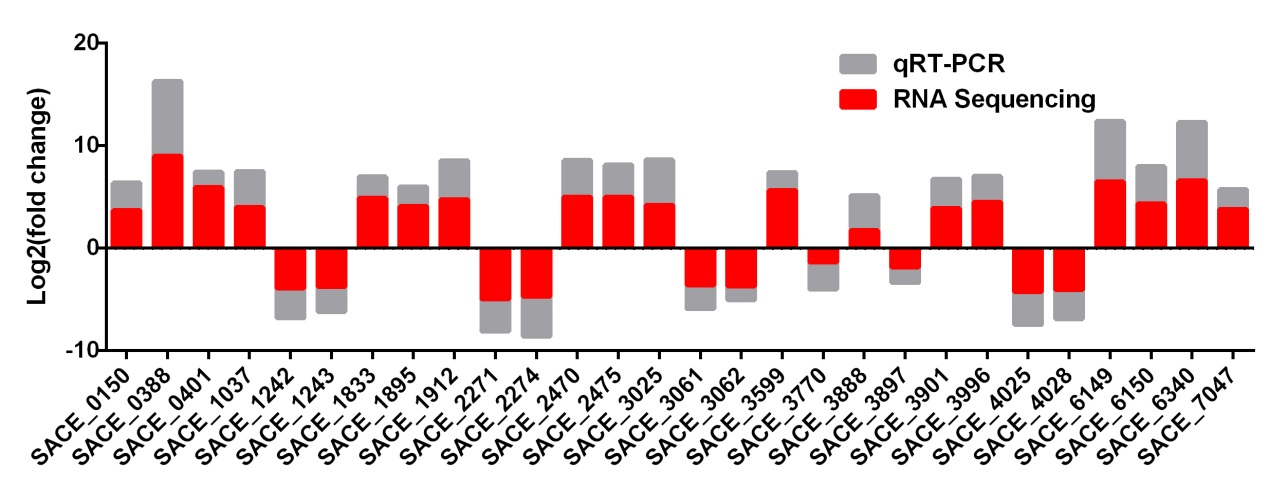
**

**Figure S7 qRT-PCR analysis of the accuracy of transcriptome analysis.** Gray bars indicate the relative expression levels by transcriptome sequencing and red bars represent the relative expression levels by qRT-PCR. All relative expression values were shown with log2 ^(Δ^*^SACE_5754^*^/A226)^ on the Y axis.

1. Wu H, Wang Y, Yuan L, Mao Y, Wang W, Zhu L, Wu P, Fu C, Müller R, Zhang L, et al: **Inactivation of SACE_3446, a TetR family transcriptional regulator, stimulates erythromycin production in *Saccharopolyspora erythraea*.** *Synthetic and Systems Biotechnology* 2016, **1:**39-46.

2. Han S, Song P, Ren T, Huang X, Cao C, Zhang B: **Identification of SACE_7040, a member of TetR family related to the morphological differentiation of *Saccharopolyspora erythraea*.** *Curr Microbiol* 2011, **63:**121-125.

3. Liu J, Chen Y, Wang W, Ren M, Wu P, Wang Y, Li C, Zhang L, Wu H, Weaver DT, Zhang B: **Engineering of an Lrp family regulator SACE_Lrp improves erythromycin production in *Saccharopolyspora erythraea*.** *Metab Eng* 2017, **39:**29-37.

4. Wilkinson CJ, Hughes-Thomas ZA, Martin CJ, Bohm I, Mironenko T, Deacon M, Wheatcroft M, Wirtz G, Staunton J, Leadlay PF: **Increasing the efficiency of heterologous promoters in actinomycetes.** *J Mol Microbiol Biotechnol* 2002, **4:**417-426.
